# Transcriptomic alterations in APP/PS1 mice astrocytes lead to early postnatal axon initial segment structural changes

**DOI:** 10.1007/s00018-024-05485-9

**Published:** 2024-11-01

**Authors:** María José Benitez, Diana Retana, Lara Ordoñez-Gutiérrez, Inés Colmena, María José Goméz, Rebeca Álvarez, María Ciorraga, Ana Dopazo, Francisco Wandosell, Juan José Garrido

**Affiliations:** 1 Instituto Cajal, CSIC, Madrid, Spain; 2 Alzheimer’s Disease and Other Degenerative Dementias, Centro de Investigación Biomédica en Red de Enfermedades Neurodegenerativas (CIBER-ISCIII), Madrid, Spain; 3 Departamento de Química Física Aplicada, Universidad Autónoma de Madrid, Madrid, Spain; 4 Centro de Biología Molecular “Severo Ochoa” (CSIC-UAM), Universidad Autónoma de Madrid, Madrid, Spain; 5 Genomics Unit, Centro Nacional de Investigaciones Cardiovasculares (CNIC), Madrid, Spain

**Keywords:** Axon initial segment, Astrocytes, Neurodegeneration, ADNP, AnkyrinG, Retinoic acid, P2X7

## Abstract

Alzheimer´s disease (AD) is characterized by neuronal function loss and degeneration. The integrity of the axon initial segment (AIS) is essential to maintain neuronal function and output. AIS alterations are detected in human post-mortem AD brains and mice models, as well as, neurodevelopmental and mental disorders. However, the mechanisms leading to AIS deregulation in AD and the extrinsic glial origin are elusive. We studied early postnatal differences in AIS cellular/molecular mechanisms in wild-type or APP/PS1 mice and combined neuron-astrocyte co-cultures. We observed AIS integrity alterations, reduced ankyrinG expression and shortening, in APP/PS1 mice from P21 and loss of AIS integrity at 21 DIV in wild-type and APP/PS1 neurons in the presence of APP/PS1 astrocytes. AnkyrinG decrease is due to mRNAs and protein reduction of retinoic acid synthesis enzymes Rdh1 and Aldh1b1, as well as ADNP (Activity-dependent neuroprotective protein) in APP/PS1 astrocytes. This effect was mimicked by wild-type astrocytes expressing ADNP shRNA. In the presence of APP/PS1 astrocytes, wild-type neurons AIS is recovered by inhibition of retinoic acid degradation, and Adnp-derived NAP peptide (NAPVSIPQ) addition or P2X7 receptor inhibition, both regulated by retinoic acid levels. Moreover, P2X7 inhibitor treatment for 2 months impaired AIS disruption in APP/PS1 mice. Our findings extend current knowledge on AIS regulation, providing data to support the role of astrocytes in early postnatal AIS modulation. In conclusion, AD onset may be related to very early glial cell alterations that induce AIS and neuronal function changes, opening new therapeutic approaches to detect and avoid neuronal function loss.

**Supplementary Information:**

The online version contains supplementary material available at 10.1007/s00018-024-05485-9.

## Introduction

The main characteristic of Alzheimer´s disease (AD) is progressive neurodegeneration and cognitive impairment that appear without an obvious triggering cause in 95% of the cases. AD's main clinical hallmarks are the progressive accumulation of β-amyloid plaques and neurofibrillary tangles. APP(Swe^695^)/PS1(ΔE9) mice are characterized by the appearance of very small β-amyloid plaques at 4 months of age in the cortex, and plaques are evident in the cortex and hippocampus at 6 months of age. However, these hallmarks appear also in brains without Alzheimer´s symptoms [1], and high Aβ42 levels are proposed as predictors of normal cognition in individuals with AD-causing genetic mutations [2]. The initial cellular and molecular mechanisms leading to AD neurodegeneration are elusive. Recent hypotheses postulate that AD onset may be due to (1) early alterations in neuronal excitability balance mechanisms and associated neuronal structures or (2) neuroinflammatory mechanisms mediated by changes in glial cells, leading to later neurodegeneration and cognitive impairment.

Excitation/Inhibition (E/I) imbalance occurs early in preclinical AD and influences the accumulation rate of protein deposition [3, 4]. Increased incidence of seizures is highest in cases with early onset in sporadic AD patients [5]. In this context, the axon initial segment (AIS) is the neuronal domain where excitatory and inhibitory neuronal inputs are integrated to generate the action potentials [6] and represents a potential target of AD onset mechanisms. The AIS contains a complex structure of specific structural scaffold proteins that maintain the AIS, such as ankyrinG, βIV-spectrin, or PSD-93. These scaffold proteins anchor membrane proteins, such as neurofascin, and a high density of voltage-gated ion channels, such as Na_v_1, K_v_1.1, or K_v_7.2/3, that contribute to action potential generation [6, 7]. This entire complex is bound to AIS microtubules cytoskeleton by ankyrinG through EB1/3 proteins, while βIV-spectrin binds this complex to actin microfilaments. Besides, the AIS is characterized by a high degree of structural plasticity allowing AIS adaptation to global neuronal input to modulate neuronal output [8, 9]. Changes in different monoamines, purines, or cannabinoid receptors alter AIS composition or structural plasticity [10–12]. AIS disruption happens before neuronal death [13], while partial loss of AIS integrity leads to abnormal neuronal excitability and comprises neuronal polarity [10, 13, 14], altering the communication between neurons and rendering the neuron dysfunctional.

Previous studies reported AIS alterations in adult AD mouse models and human AD brains [15, 16]. APP/PS1 brains show decreased expression of sodium channels at very early postnatal stages [17]. Interestingly, βIV-spectrin epigenetic deregulation and mRNA downregulation happen in APP/PS1 mice and AD patients [18]. Decreased action potential firing frequency was found in 1 month APP/PS1 mice [19], which do not show amyloid plaques and very low Aβ40 levels. In young APP/PS1 mice, it was described a reduced expression of voltage-gated sodium channels [17], while older Tg2576 (APPSwe) mice show increased sodium channel expression and hyperexcitability [20]. In addition, APP or APP fragments regulate or decrease the activity of Kv7 potassium voltage-gated ion channels, known to be concentrated at the AIS and nodes of Ranvier [21]. Regarding AIS plasticity, AISs are shorter around βA plaques in aged mice from different models of Alzheimer´s disease [15], while tau-mediated distal relocation of the AIS in Alzheimer´s mice model expressing tauP301L led to suppression of hippocampal excitability [22]. Moreover, in a frontotemporal dementia model, a TauV337M mutation in human neurons derived from iPSCs impairs axon initial segment plasticity [23]. AIS alterations, such as protein composition or length changes, are also a characteristic identified in other neurodevelopmental diseases, brain trauma, or mental disorders [10, 24–26].

Early neuronal excitability alterations may also result from glial cells alterations. Glial cells are major actors of brain neuroinflammation and are proposed as mediators of the mechanisms related to Alzheimer´s disease onset [27]. Astrocytes, the most abundant glial cells, are responsible for brain homeostasis and become reactive in pathological conditions losing many of their neuroprotective functions [28]. Astrocyte activation occurs early, even before the mild cognitive impairment (MCI) phase, and reaches a plateau when clinical symptoms appear [29]. Together with astrocytes, microglial cells complete a neuroinflammatory loop amplifying the neurodegenerative cycle [30]. Microglia activation seems to have a neuroprotective effect in early AD stages but becomes harmful in further disease stages [31]. Morphological changes and mRNA expression modifications are concomitant to astrocytes and microglia activation in AD post-mortem human brains and AD mice models [32, 33]. However, it is unknown whether early postnatal astrocyte alterations may affect neurons and induce their loss of function and neurodegeneration in AD.

In this context, we analyzed the integrity of the axon initial segment in control and APP/PS1 mice from 15 days postnatal mice (P15) to 16 months mice. Our results show reduced ankyrinG expression at the AIS from 21 days in APP/PS1 mice and a reduction of AIS length. We confirmed these results in co-cultured neurons and astrocytes and identified APP/PS1 astrocytes as the factor contributing to AIS loss of integrity. Comparative RNAseq analysis of wild-type and APP/PS1 astrocytes identified mRNA changes, among them, a reduction of mRNAs coding for ADNP (Activity-Dependent Neuroprotective Protein), Rdh1 (retinol dehydrogenase 1) and Aldh1b1 (Aldehyde dehydrogenase 1 Family Member B1), these last two involved in retinoic acid synthesis. AIS parameters, in the presence of APP/PS1 astrocytes, recovered to control levels through impairing retinoic acid degradation, inhibiting P2X7 function, or by the addition of the Adnp-derived peptide (NAP) to cultured medium.

## Results

### AIS composition and length modifications begin at P21 in APP/PS1 mice

**Fig. 1:**
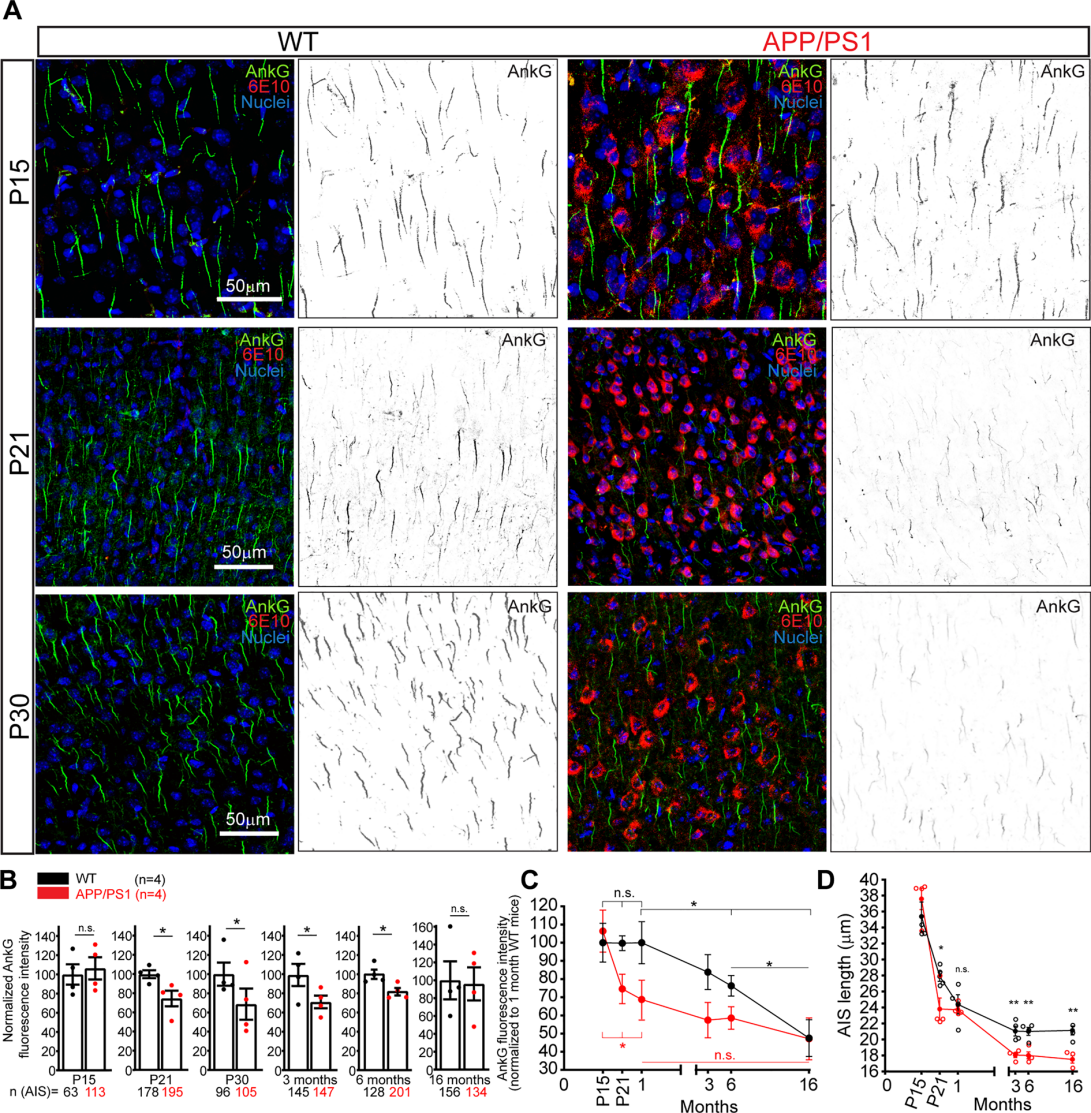
Early loss of ankyrinG density and AIS shortening in P21 APP/PS1 mice cortex. **A** Representative images of somatosensory cortex S1 region brain sections of wild-type or APP/PS1 mice compared in the same litters at postnatal day 15 (P15), 21 (P21), or 30 (P30). Brain sections were stained with antibodies against ankyrinG (AnkG, green) and β-amyloid (6E10, red). Nuclei were stained using bisbenzimide H33342 (blue). Inversed greyscale images show ankyrinG staining at each age. **B** Graphs show ankyrinG fluorescence intensity in WT (black) and APP/PS1 (red) mice (n = 4) normalized to the corresponding WT mice at different brain ages. **C** Normalized ankyrinG fluorescence intensity in WT (black) and APP/PS1 (red) mice normalized to P30 WT mice (n = 4). P30 WT sections were used as a reference in all immunofluorescence studies. **D** AIS length in WT (black) or APP/PS1 (red) mice at different ages (n = 4). Scale bar = 50 μm. Each data point represents the mean ± SEM of 4 mice (open symbols) and the total number of AISs is shown in B at the bottom for each point. *p < 0.05, n.s. (not significant), Mann–Whitney test

To determine whether and when the AIS is altered in an AD mice model, we first analyzed the AIS composition and length of cortical and hippocampal neurons in APP/PS1 or WT mice at different life stages between postnatal P15 and 16 months (Fig. 1A and Supplementary Fig. 1). Analysis of ankyrinG (AnkG) fluorescence intensity on cortical neurons AISs show no difference in P15 APP/PS1 mice (Fig. 1A, B) compared to WT mice (n = 4). However, a significant AnkG intensity reduction, around 30%, was identified in P21 APP/PS1 mice (74.5.9 ± 8.1%) compared to WT animals in the same litter (100 ± 2.4%) and was maintained in P30 APP/PS1 mice (68.72 ± 16.2% *vs* 100 ± 11.9%, Fig. 1B). The same AnkG reduced intensity was maintained at 3 months and 6 months in APP/PS1 mice compared to P30 WT mice (71.05 ± 6.6% and 81.8 ± 3.8%, Fig. 1B). However, our data show that AnkG intensity decreased progressively in 3 and 6-month WT mice compared to P30 WT mice (83.8 ± 9.6%, 76.3 ± 5.7%), suggesting a relation between AnkG intensity and WT mice age increase. AnkG intensity was only 47.5 ± 10.1% in 16 months WT mice compared to P30 WT mice (Fig. 1C). AnkG intensity decreases in APP/PS1 mice compared to WT was around 20–30% at 3 and 6 months (Fig. 1B). However, there was no significant difference between WT and APP/PS1 mice at 16 months (Fig. 1B, C).

**Fig. 2:**
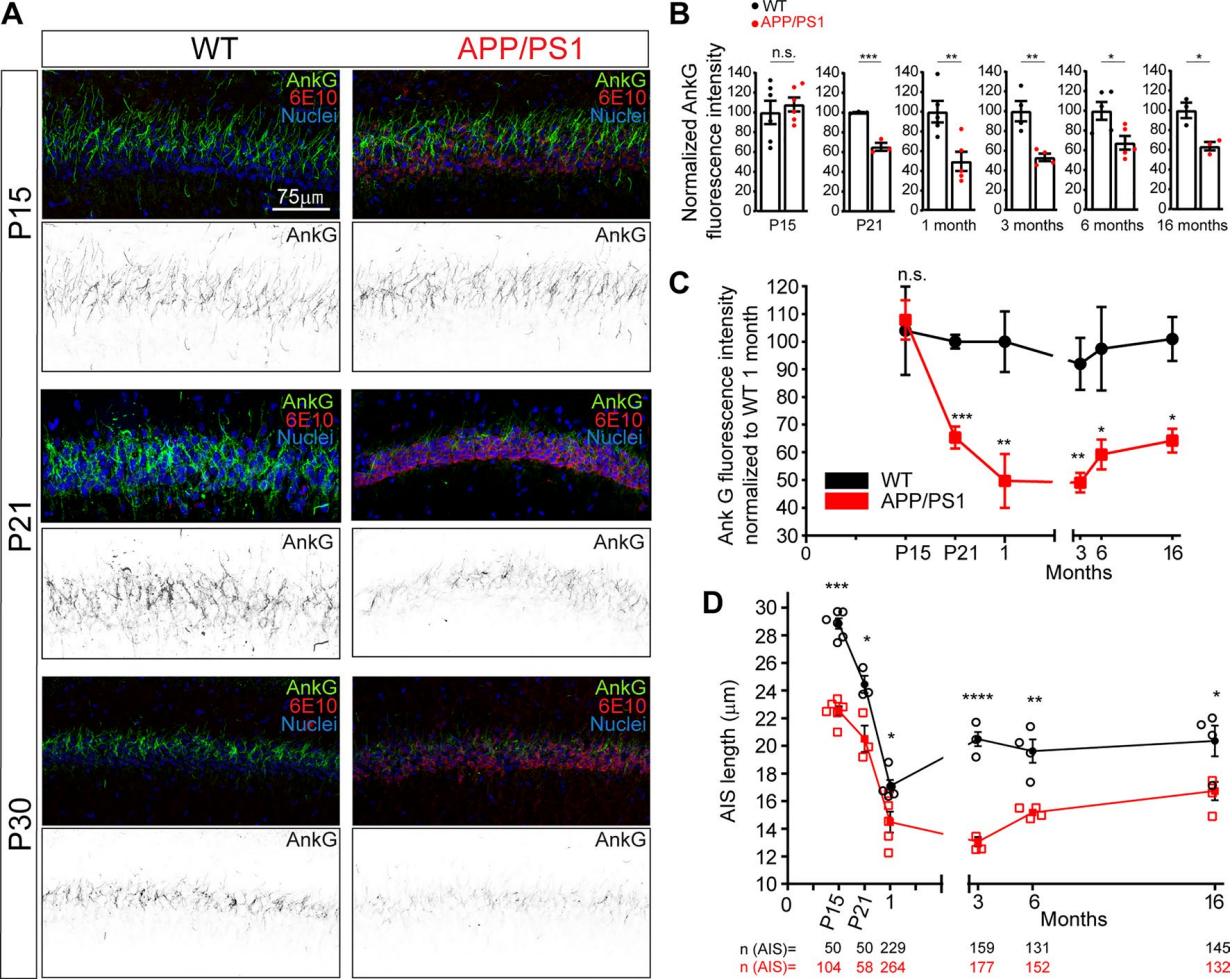
Early loss of ankyrinG density and AIS shortening in P21 APP/PS1 mice hippocampus **A** Representative images of CA1 hippocampal sections of wild-type or APP/PS1 mice compared in the same litters at postnatal day 15 (P15), 21 (P21) or 30 (P30). Brain sections were stained with antibodies against ankyrinG (AnkG, green) and β-amyloid (6E10, red). Nuclei were stained using bis-benzimide (blue). Inversed greyscale images show ankyrinG staining at each age. **B** Graphs show ankyrinG fluorescence intensity in CA1 region of WT mice (black points) and APP/PS1 (red points) mice normalized to the corresponding WT mice at different brain ages. **C** AnkyrinG fluorescence intensity in WT (black) and APP/PS1 (red) mice normalized to P30 WT mice. P30 WT sections were used in all immunofluorescence studies as a reference. Data were obtained from 3 to 5 WT or APP/PS1 mice from the same litter and analyzing three CA1 sections in each mouse. **D** AIS length in WT (black circles) or APP/PS1 (red squares) mice at different ages. Each data point represents the mean ± SEM of the corresponding number of mice (open symbols). Total number of AISs is shown at the bottom. Scale bar = 75 μm. *p < 0.05, **p < 0.01, ***p < 0.001, ****p < 0.0001. Mann–Whitney test

Due to the importance of AIS structural plasticity in physiological and pathological conditions, we next examined potential AIS length differences, based on the AnkG intensity profile (Fig. 1D). In WT mice, AIS length decreased from P15 to 3 months (35.3 ± 1.8 μm at P15, 27.9 ± 0.6 μm at P21, 24.4 ± 1.2 μm at P30, and 21.05 ± 0.6 μm at 3 months), and then AIS maintained its length till 16 months (21.1 ± 0.6 μm). In APP/PS1 mice, no differences were detected at P15 (37.5 ± 1.3 μm) compared to WT mice, but AIS was significantly shorter (≈15%) at P21 in APP/PS1 mice (23.8 ± 1.4 μm *vs* 27.9 ± 0.6 μm in WT mice). There was no difference at P30 between WT and APP/PS1 mice (24.4 ± 1.2 μm *vs* 23.7 ± 0.37 μm), but a significant difference was found again at 3 months (18.04 ± 0.3 μm *vs* 21.05 ± 0.6 μm, ≈ 15%). AIS shortening was maintained at 6 months (18.01 ± 0.45 μm *vs* 21 ± 0.5 μm, ≈ 15%) and 16 months (17.5 ± 0.45 μm *vs* 21.1 ± 0.6 μm, ≈ 17%). In parallel, we examined the same parameters in the CA1 region of the hippocampus (Fig. 2A and Supplementary Fig. 1). As measured in the cortex, no AnkG intensity differences were found at P15 between WT and APP/PS1 mice (Fig. 2A, B). However, AnkG intensity decreased by around 35% in P21 APP/PS1 mice (65.3 ± 4%) and around 50% in P30 APP/PS1 mice (49.7 ± 9.7%). This reduction was similar in 3 months (53.3 ± 3.9%), 6 months (67.6 ± 6.9%), and 16 months (63.6 ± 4.2%) APP/PS1 mice compared to their respective WT mice. Unlike the constant AnkG decrease observed in WT mice cortex from birth to 16 months, ankyrinG rested at the same level as observed at P15 in WT mice, while APP/PS1 mice from P21 show a significant decrease when normalized to P30 WT mice data (Fig. 2C). Regarding AIS length (Fig. 2D), AIS shortening was detected in WT mice from P15 to 3 months (28.8 ± 0.37 μm at P15, 24.3 ± 0.5 μm at P21, 17 ± 0.14 μm at P30 and 20.5 ± 0.2 μm at 3 months), maintaining this length at 6 and 16 months. Meanwhile, APP/PS1 mice AIS length was significantly shorter at each age (P15, 22%; P21, 16%; P30, 15%) and more pronounced at 3 months (36%) than their WT littermates. AIS was also shorter at 6 and 16 months (23% and 18%, respectively).

### AnkyrinG expression decreases in the axon initial segment of APP/PS1 cultured hippocampal neurons at 21 DIV

**Fig. 3:**
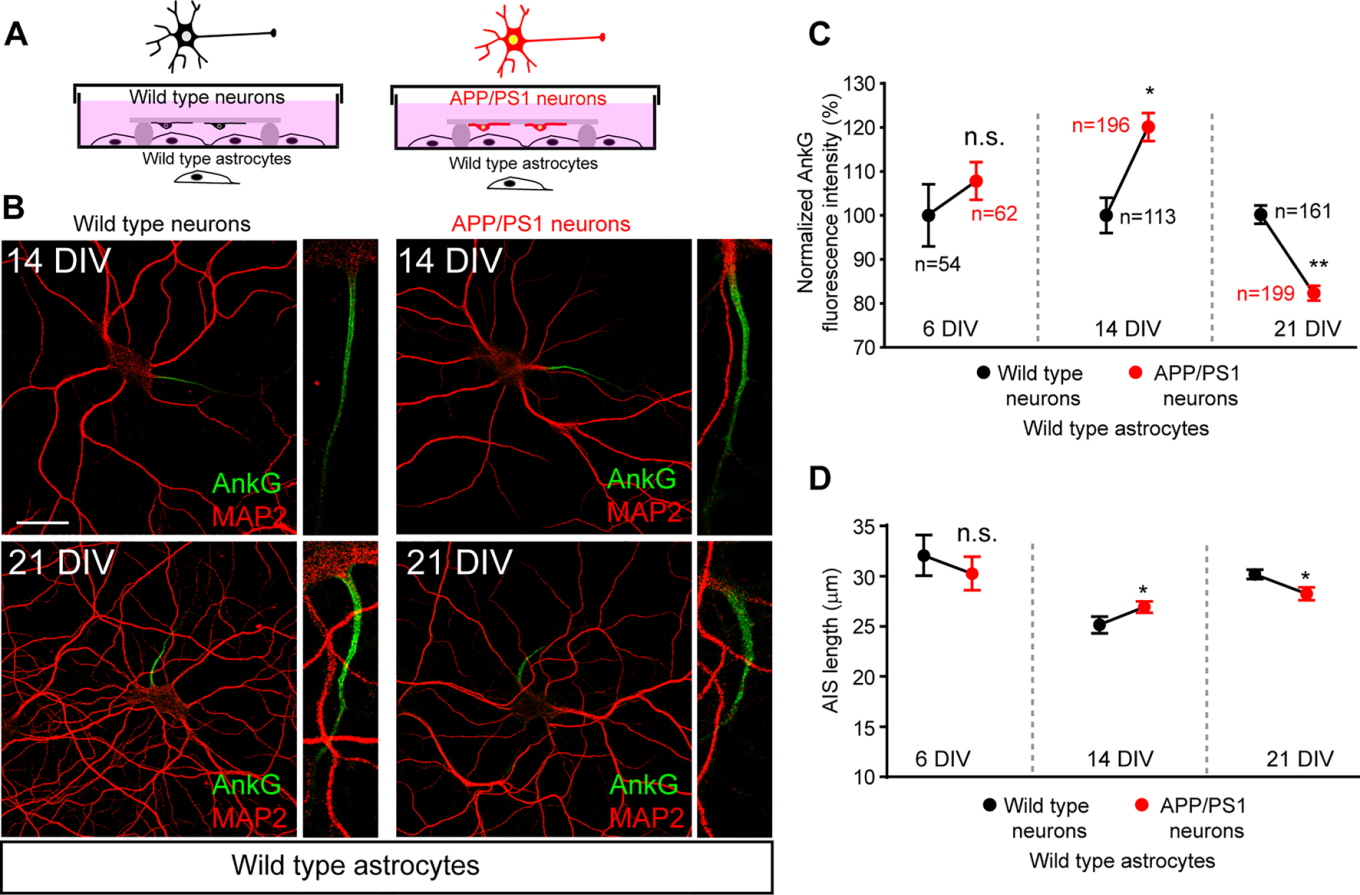
AnkyrinG expression in cultured APP/PS1 hippocampal neurons. **A** Schematic representation of the culture of WT and APP/PS1 hippocampal neurons in the presence of WT astrocytes. Hippocampal neurons were obtained from the same litter and the genotype was revealed after quantifications. Neurons were cultured during 6, 14, or 21 DIV. **B** Representative images of 14 and 21 DIV hippocampal neurons (WT or APP/PS1) cultured in the presence of WT astrocytes. Neurons were stained with antibodies against MAP2 (red) and ankyrinG (green). Scale bar = 25 μm. **C** Normalized ankyrinG density in 6, 14, and 21 DIV WT and APP/PS1 neurons. Data represents the mean ± SEM of the number of neurons indicated in the graph and obtained from three independent experiments containing each one 6, 14, and 21 DIV neurons. *p < 0.05, **p < 0.01, n.s. (not significant), Mann–Whitney test. **D** Mean ± SEM AIS length of neurons represented in C. *p < 0.05, (*n.s.* not significant), Mann–Whitney test

The above results show early postnatal modifications of AIS in APP/PS1 mice beginning at P21. To decipher the mechanisms and due to the parallelism observed in neuronal development “in vivo” and “in vitro” [34], we analyzed AIS development and maturation at 6, 14, and 21 DIV in WT and APP/PS1 cultured hippocampal neurons from the same litters (Fig. 3) in the presence of WT astrocytes (Fig. 3A, B). Immunofluorescence using the 6E10 antibody against β-amyloid (Supplementary Fig. 2A) further confirmed the neuron's genotype. AnkG fluorescence intensity was measured at 6, 14, and 21 DIV along the AIS (Fig. 3C). Our data show no significant changes in AnkG fluorescence intensity in APP/PS1 at 6 DIV (107.8 ± 4.26%), a significant increment of 20% at 14 DIV (120 ± 3.1%), and a significant decrease around 20% at 21 DIV (80.7 ± 1.2%) compared to 100% AnkG intensity in WT neurons. Same changes were also found for AIS length in 14 (26.8 ± 0.56 μm *vs* 25.05 ± 0.83 μm) and 21 (28.04 ± 0.63 μm *vs* 29.98 ± 0.45 μm) DIV APP/PS1 neurons (Fig. 3D). These data confirm the results obtained in brain sections and suggest that mutated human APP/PS1 expression in neurons contributes to AnkG decrease during the final stage of AIS maturation. However, it is not possible to discard a role of astrocytes due to their interaction with neurons and the fact that ankyrinG decrease happens only around 21 DIV.

### APP/PS1 astrocytes alter axon initial segment composition and length

**Fig. 4:**
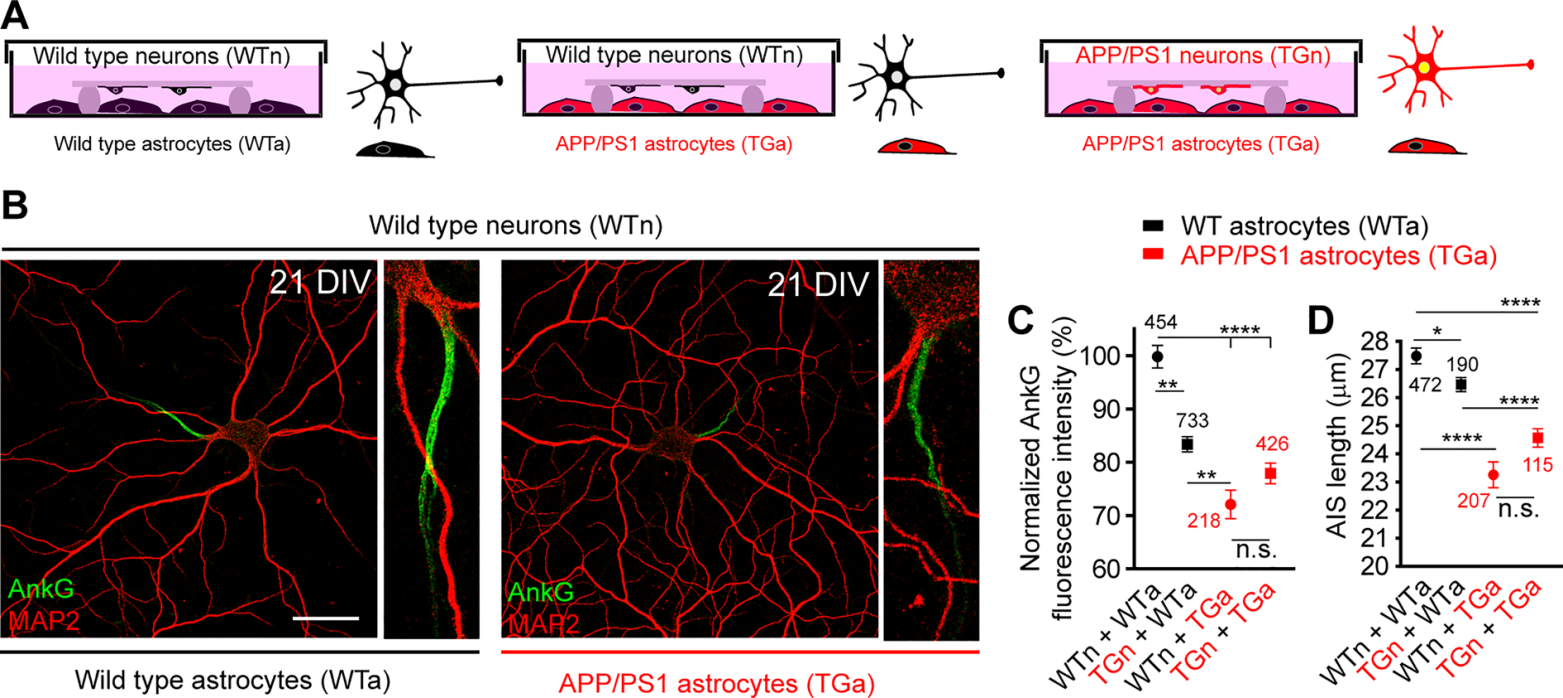
APP/PS1 astrocytes decrease ankyrinG and AIS length of WT and APP/PS1 neurons. **A** Schematic representation of the culture of WT and APP/PS1 hippocampal neurons in the presence of WT or APP/PS1 astrocytes. Hippocampal neurons were obtained from the same litter and the genotype was revealed after quantifications. Neurons from every embryo were cultured in the presence of WT or APP/PS1 astrocytes for 21 DIV. **B** Representative images of wild-type 21 DIV hippocampal neurons cultured in the presence of WT or APP/PS1 astrocytes. Neurons were stained with antibodies against MAP2 (red) and ankyrinG (green). Scale bar = 25 μm. **C** Normalized ankyrinG density in 21 DIV WT or APP/PS1 neurons cultured in the presence of WT or APP/PS1 astrocytes. Data were normalized to WT neurons and WT astrocytes co-culture. **D** AIS length in different WT and APP/PS1 neurons or astrocytes combinations. Data number for each condition is indicated together with their mean ± SEM. Data were obtained from at least 5 independent experiments. **p < 0.01, ***p < 0.001, p < 0.0001, n.s. (not significant). 2-way ANOVA test followed by Bonferroni test for multiple comparisons. 2-way ANOVA analysis for AnkyrinG intensity or AIS length (GraphPad) shows an extremely significant effect of astrocytes genotype (p < 0.0001 and p < 0.0001), while neurons genotype effect is considered not significant (p = 0.1767 and p = 0.6688, respectively)

We confirmed that APP/PS1 astrocytes show 6E10 staining (Supplementary Fig. 2B, C) and their culture medium contained Aβ1-40 peptides (112.8 ± 31 pg/ml) and Aβ1-42 (168 ± 61 pg/ml), not detected in WT astrocytes medium. Then, we cultured WT or APP/PS1 neurons till 21 DIV in the presence of WT or APP/PS1 astrocytes from the same litter (Fig. 4A, B). Our results show that AnkG decreased around 30% in WT neurons in the presence of APP/PS1 astrocytes (Fig. 4C), suggesting a relevant role of APP/PS1 astrocytes in AnkG intensity reduction. Only a 15% decrease was observed in APP/PS1 neurons in the presence of WT astrocytes. No significant differences were observed between WT and APP/PS1 neurons in the presence of APP/PS1 astrocytes (Fig. 4C). Two-way ANOVA analysis comparing astrocytes and neuronal variables renders a significant effect for astrocytes genotype (p < 0.0001), while the neuron genotype does not have a significant role (p = 0.1767). Concerning AIS length (Fig. 4D), there was a slight significant difference in AIS length between WT and APP/PS1 neurons in the presence of WT astrocytes (27.5 ± 0.3 μm vs 26.5 ± 0.2 μm, respectively, p = 0.046). However, AIS length was significantly reduced in both neuronal genotypes in the presence of APP/PS1 astrocytes (23.2 ± 0.4 μm and 24.5 ± 0.3 μm *vs* 27.4 ± 0.3 μm in WT neurons with WT astrocytes, p < 0.0001). These results point to APP/PS1 astrocytes as a significant source of factors contributing to AIS composition and structure alterations. Thus, we examined potential genetic or physiological alterations in APP/PS1 astrocytes that could produce these AIS alterations.

### Transcriptomic alterations in APP/PS1 cultured astrocytes

**Fig. 5:**
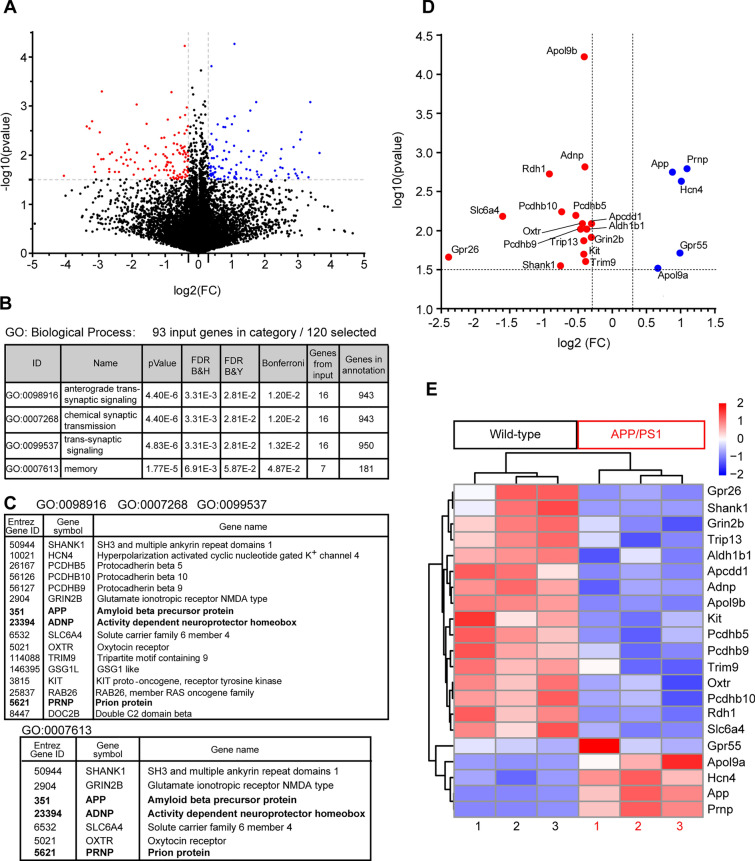
Comparative transcriptomic analysis between wild-type and APP/PS1 astrocytes. **A** Volcano plot showing the global transcriptional changes in APP/PS1 astrocytes *vs* wild-type astrocytes. All genes identified were plotted. Each circle represents one gene. The log fold change (log2(FC)) in APP/PS1 genotype versus wild type is represented on the x-axis. The y-axis shows the log10 of the p value. A p-value of 0.03 (log10(p-value) = 1.5) and a log2(fold change) of 0.3 and – 0.3 of 2 are indicated by grey dotted lines. Up-regulated and down-regulated genes are shown in blue and red, respectively. Black points represent genes without a significative p-value and a log2(FC) between -0.3 and 0.3. **B** GO analysis of genes shown in red and blue in A. **C** Tables show the genes found in each biological process category after gene ontology analysis. **D** Volcano plot showing the transcriptional changes in genes identified in GO analysis and other genes with a significative change. **E** Heatmap showing the normalized expression of genes shown in D, in each one of the three WT or APP/PS1 mice used to obtain cultured astrocytes

Due to the role of APP/PS1 astrocytes in AIS alterations, we performed a transcriptomic analysis of APP/PS1 and WT astrocytes to find potential expression differences. Astrocytes were obtained from 3 WT and 3 APP/PS1 P0 mice and were cultured for 15 DIV as previously described [35]. Isolated RNA was used to perform RNA-seq determination as described in Methods, and expression of the most representative genes in each mouse WT or APP/PS1 was represented in a heatmap (Fig. 5E). A total of 21,431 genes were analyzed and normalized mRNA expression modifications were plotted in a Volcano plot graph (Fig. 5A, https://saco.csic.es/index.php/s/EZqD7EEYfPpM3fC). We selected those upregulated or downregulated genes with a log2(FC) ≥ 0.3 or ≤ -0.3 and – log10(p-value) ≥ 1.5, representing a total of 120 genes (Supplementary Table 1). ToppGene analysis of Biological processes revealed some clusters of genes associated with synaptic signaling and memory (Fig. 5B, C). Analysis of these genes through Gene ontology (GO) software rendered a cluster of 16 genes involved in synaptic signaling and a cluster of 7 genes involved in memory (Fig. 5C). APP and PrP mRNA were used as internal controls showing a significant upregulation in APP/PS1 astrocytes (Fig. 5D). Some genes involved in cellular homeostasis, such as Hcn4, were upregulated, as well as the cannabinoids receptor Gpr55 or Apol9a (Apolipoprotein L9a). The downregulated gene with a higher significance (log2(FC) = – 0.41 and – log10(p-value) = 4.22) was Apolipoprotein L9b (Apol9b), which function is unknown, is not secreted and can be induced by interferon [36]. Interestingly, a secreted protein related to Alzheimer´s disease and AIS proteins EB1/3 [37, 38], the Activity-Dependent Neuroprotective Protein (ADNP), was downregulated (log2(FC) = – 0.40 and – log10(p-value) = 2.81). Interestingly, oxytocin receptor mRNA was also downregulated and previous results showed a role of astrocytes in oxytocin-mediated control of neural circuits [39]. Moreover, oxytocin increases VIP levels, which modulate Adnp expression [40]. We found also decreased mRNA expression of the serotonin transporter Slc6a4/5-HTT (solute carrier family 6 member 4) suggesting increased serotonin levels in medium and higher activation of 5-HT1A receptor, which play a role in AIS regulation [41, 42]. Among the 120 genes, we also found two downregulated retinoic acid synthesis enzymes, Rdh1 and Aldh1b1 (Fig. 5D).

### Alterations of retinoic acid synthesis decrease AIS length and ankyrinG density

**Fig. 6:**
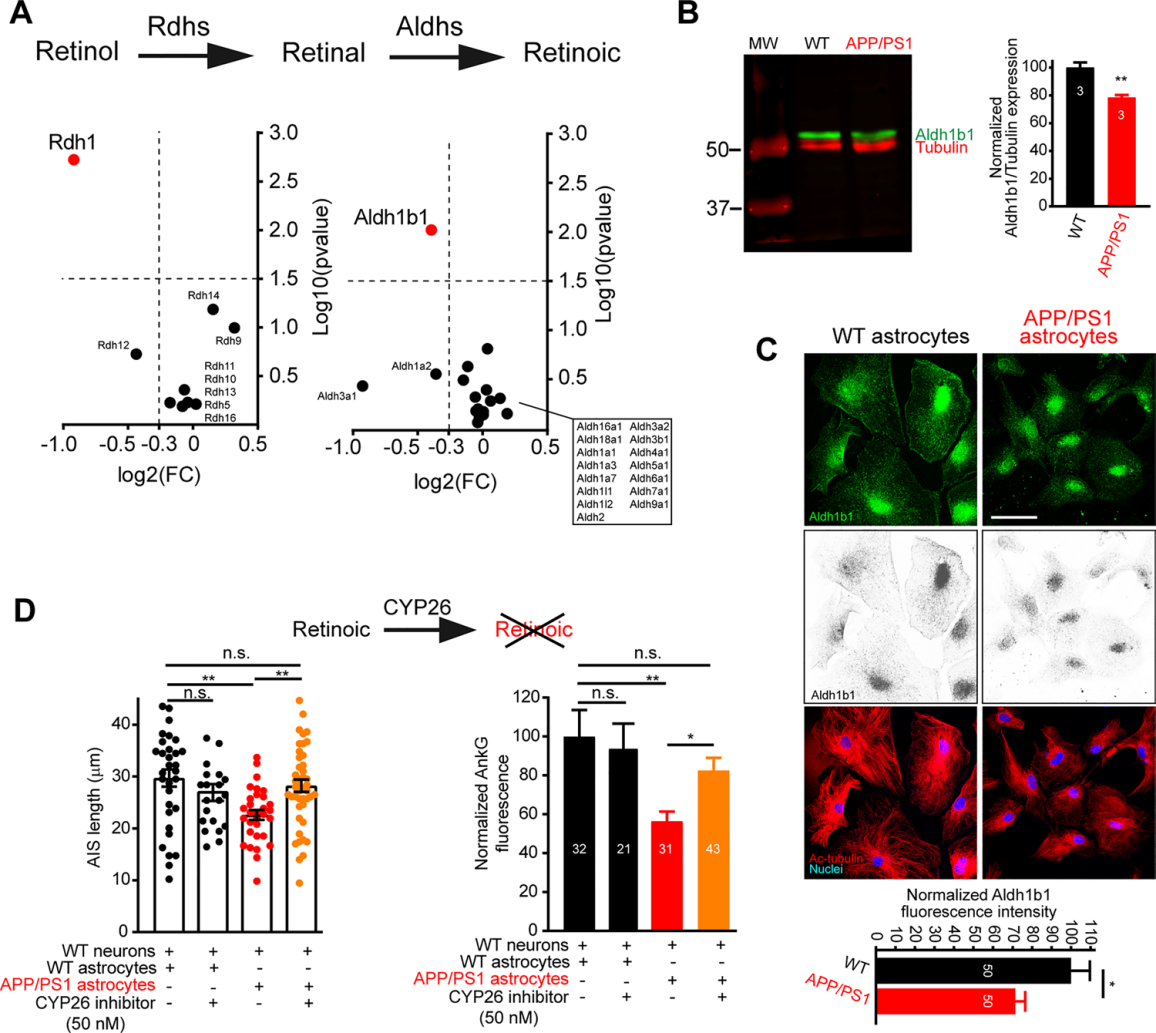
Retinoic acid synthesis enzymes downregulation in APP/PS1 astrocytes contribute to axon initial segment alterations. **A** Volcan plots of Rdhs and Aldhs genes identified in astrocytes. Rdh1 and Aldh1b1 are shown in red and are the only ones to significantly change their expression. All other Rdhs and Aldhs genes are shown in black and named in the graph. **B** Western-blot showing Aldh1b1 (green) expression in WT and APP/PS1 astrocytes in culture. Data are normalized to their respective tubulin levels (red) in 3 independent experiments. *p < 0.01, t-test. **C** Images show Aldh1b1 expression (green or greyscale). Astrocytes morphology was identified by acetylated tubulin staining (red) and nuclei are shown in blue. Scale bar = 100 μm. The graph represents the normalized Aldh1b1 protein expression quantified by immunofluorescence in 50 WT astrocytes and 50 APP/PS1 astrocytes obtained from 3 WT or APP/PS1 mice. *p < 0.05, Mann–Whitney test. **D** Axon initial length and normalized ankyrinG intensity in WT neurons co-cultured with WT astrocytes or APP/PS1 astrocytes, and treated with vehicle or an inhibitor of CYP26 enzyme for 3 days. Each condition represents the mean ± SEM of the neurons indicated in panel D obtained from 3 independent experiments. *p < 0.05, **p < 0.01. 2-way ANOVA and multiple comparisons Bonferroni test.

We found mRNA decrease of 2 out of 28 enzymes involved in retinoic acid synthesis detected in astrocytes, the retinol dehydrogenase mRNA (Rdh1) and the aldehyde dehydrogenase (Aldh1b1), which act in sequence to generate retinoic acid (Fig. 6A). Interestingly, defective retinoid functions have been involved in the late onset of Alzheimer´s disease [43]. In the absence of specific Rdh1 antibodies, we checked the expression of Aldh1b1 in astrocytes by Western-blot or immunofluorescence (Fig. 6B, C). APP/PS1 astrocytes show lower expression of Aldh1b1 protein (22% or 30%, respectively) compared to WT astrocytes, confirming results obtained from mRNA analysis. A previous study confirmed that retinoic acid accumulation in neurons is significantly much lower than in astrocytes, but increases the amount in hippocampal neurons in the presence of astrocytes co-culture, demonstrating that neurons sequester retinoic acid secreted by astrocytes [44]. The study shows that neurons have low if any retinol and retinal dehydrogenase activities.

To further confirm the potential role of retinoic acid in AIS alterations, we inhibited CYP26, the enzyme responsible for degrading retinoic acid, using a selective inhibitor of CYP26 (50 nM), to preserve retinoic acid levels. Actually, knockdown of Cyp26B1 by siRNA in astrocytes results in a fivefold increase in retinoic acid levels [44]. CYP26 expression in rodent neurons has not been described, although one study in adult human brain hippocampus sections postulate the expression of CYP26B1 in some neurons [45]. We analyzed ankyrinG and AIS length in WT hippocampal neurons cultured in the presence of WT or APP/PS1 astrocytes treated for 3 days from 19 to 21 DIV with the CYP26 inhibitor (Fig. 6D). CYP26 inhibitor did not produce any significative effect on WT neurons-WT astrocytes cultures, suggesting a saturation of retinoic acid accumulation in neurons in WT-WT cultures, as previously shown [44]. Neurons in the presence of APP/PS1 astrocytes showed a decreased ankyrinG density (56.4 ± 4.9%) and were shorter (22.6 ± 0.9 μm) compared to 100 ± 13.6% and 29.7 ± 1.6 μm, respectively, in the presence of WT astrocytes. However, CYP26 inhibitor treatment in neurons co-cultured with APP/PS1 astrocytes maintained AIS length and almost recovered ankyrinG density to WT astrocytes co-culture levels (28.2 ± 1.2 μm and 82.57 ± 6.4%, respectively) compared to neurons cultured in the presence of WT astrocytes. These results confirm a paracrine effect of astrocytes secreted retinoic acid to maintain neuronal function. Previous studies have described that retinoic acid increases Adnp expression [46], but also decreases P2X7 expression [47], known to play a role in AIS modulation under pathological conditions [10].

### P2X7 inhibition prevents AIS alterations in APP/PS1 mice and recovers AIS in cultured neurons

Previous studies showed that inhibition of the purinergic P2X7 receptor contributes to decreasing the number of β-amyloid plaques [48], as well as, reducing glial cells mediated inflammation in APP/PS1 mice [49]. Retinoic acid decreases P2X7 expression without affecting P2X7 mRNA expression [47] and P2X7 inhibition or suppression protects and recovers AIS parameters [10]. By other hand, a P2X7 antagonist (KN-62) can reduce the increased long-term potentiation (LTP) observed in ADNP-haploinsufficient mice [50], suggesting a crosstalk between P2X7 activity and Adnp.

**Fig. 7:**
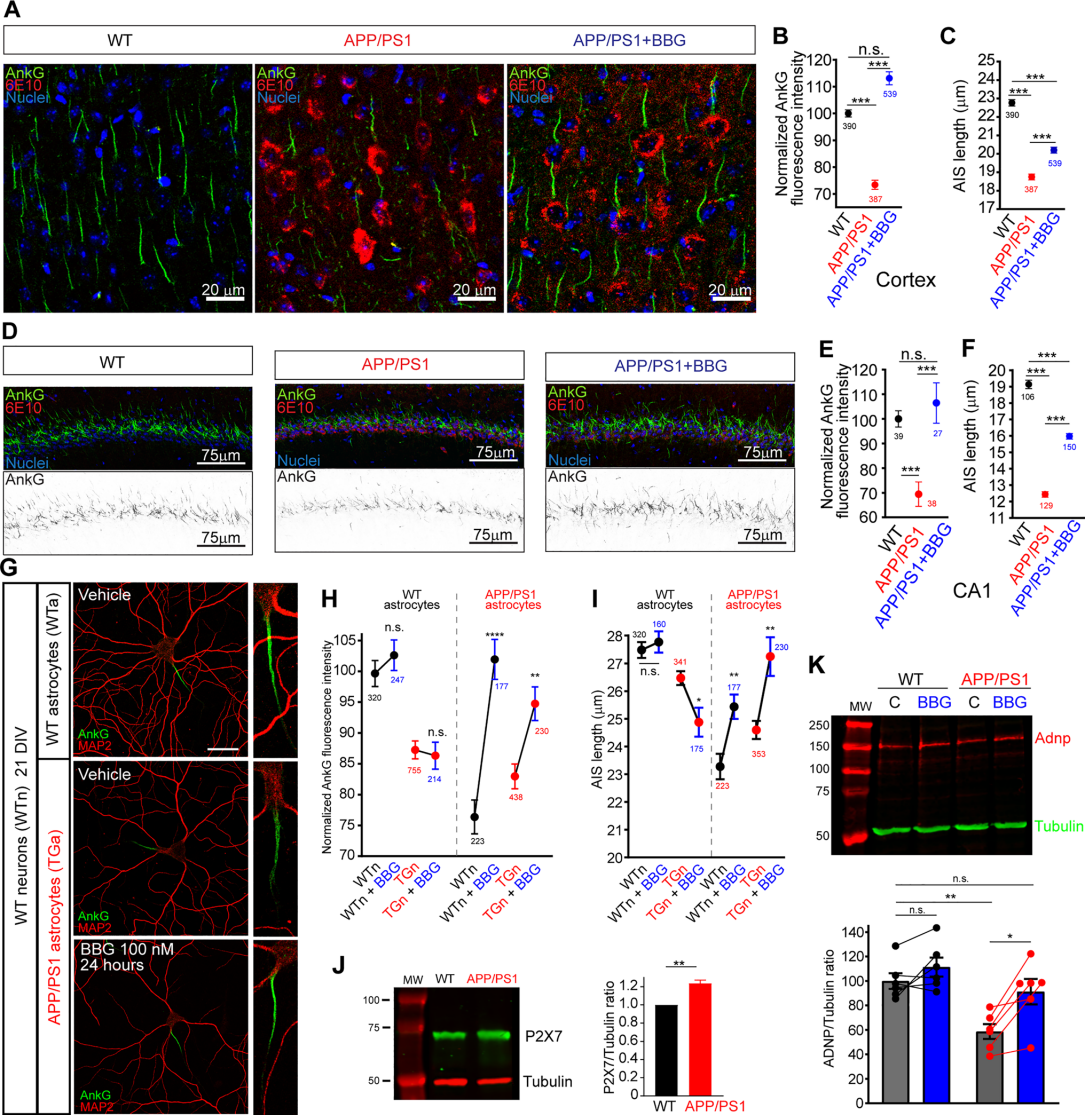
P2X7 inhibition recovers ankyrinG and AIS length in APP/PS1 mice and cultured neurons. **A**, **D** Representative images of cortical or hippocampal (CA1) sections from WT or APP/PS1 mice treated with PBS or P2X7 inhibitor BBG from 1 to 3 months. Sections were stained with antibodies against ankyrinG (green) and β-amyloid (6E10, red). Nuclei are shown in blue. Greyscale images of ankyrinG staining are shown for the hippocampus. **B**, **C** Normalized ankyrinG intensity (**C**) and AIS length (**D**) in cortical sections of WT, APP/PS1 and APP/PS1 mice treated with BBG. Data were obtained from 6 mice per condition and the number of AISs analyzed is indicated in the graphs. **E**, **F** Normalized ankyrinG intensity (**E**) and AIS length (**F**) in the hippocampus (CA1). AnkyrinG intensity in CA1 was calculated as the ankyrinG staining region. Data were obtained from 6 mice per condition and the number of CA1 sections indicated in E. AIS length was calculated from the indicated number of AISs in the CA1 region. ***p < 0.001, n.s. (not significant). One-way ANOVA and Kruskal-Willis post-hoc test with Dunn´s test multiple comparisons. **G** Representative images of 21 DIV WT neurons cultured in the presence of WT or APP/PS1 astrocytes and treated for 24 h with PBS or the P2X7 inhibitor BBG (100 nM). Neurons were stained with MAP2 (red) and AnkyrinG (green) antibodies. AISs magnifications are shown in the right panels. Scale bar = 25 μm. **H**, **I** Normalized ankyrinG (**H**) intensity and AIS length (**I**) in WT or APP/PS1 (WTn or TGn) cultured neurons in the presence of WT or APP/PS1 astrocytes treated with PBS or BBG (100 nM). Two-way ANOVA and Bonferroni test for multiple comparisons was performed in neurons cultured with WT astrocytes or in neurons cultured with APP/PS1 astrocytes. **p < 0.01, ****p < 0.0001, n.s. (not significant). **J** Representative western-blot showing P2X7 expression in cultured WT and APP/PS1 astrocytes. Graph represents the mean ± SEM of 3 independent experiments. **p < 0.01, t-test. **K** Adnp expression in WT and APP/PS1 astrocytes treated with vehicle or BBG (100 nM) for 24 h. The image shows a representative Western-blot of Adnp (red) and tubulin (green) expression. Data were obtained from 6 independent experiments and lines connect points from the same experiment. *p < 0.05, **p < 0.01, n.s. (not significant), two-way ANOVA and Bonferroni test for multiple comparisons

Therefore, we treated 1-month WT and APP/PS1 mice, every 48 h until 3 months, with intraperitoneal injections of a P2X7 receptor antagonist (BBG, 50 mg/kg) or PBS to analyze whether AIS was recovered to at least 3 months WT levels (Fig. 7). Our results show a recovery of ankyrinG in APP/PS1 mice cortex treated with BBG (111.9 ± 2.3%) compared to 100 ± 1.3% in 3 months WT mice treated with PBS and 73.4 ± 1.7% in 3 months APP/PS1 mice treated with PBS (Fig. 7A, B). AIS length was also partially recovered after BBG treatment (20.2 ± 0.16 μm) compared to PBS-treated APP/PS1 mice (18.75 ± 0.15 μm) or PBS-treated WT mice (22.7 ± 0.2 μm, Fig. 7C). AnkyrinG intensity was also recovered in the CA1 hippocampal region in APP/PS1 mice treated with BBG (106.4 ± 8.2%) compared to PBS-treated APP/PS1 mice (69.4 ± 5.0%) or WT mice treated with PBS (100 ± 3.3%, Fig. 7D, E). As observed in the cortex, the AIS length of CA1 neurons was only partially but significantly recovered in BBG-treated mice (16.0 ± 0.17 μm) compared to PBS-treated APP/PS1 mice (12.4 ± 0.17 μm) or PBS-treated WT mice (19.1 ± 0.2 μm, Fig. 7F). To confirm these results and understand the role of neurons and astrocytes in ankyrinG intensity and AIS length recovery, we added the P2X7 inhibitor, BBG (100 nM), or the vehicle (PBS) for 24 h in 21 DIV WT or APP/PS1 neurons co-cultured with WT or APP/PS1 astrocytes. The presence of APP/PS1 astrocytes reduced ankyrinG intensity by around 25% in WT neurons (76.7 ± 2.7%) that was recovered to control levels (100 ± 2.1%) after BBG (100 nM) treatment for 24 h (102.4 ± 2.4%, Fig. 7G, H). In contrast, BBG treatment did not recover ankyrinG intensity in APP/PS1 neurons co-cultured with WT astrocytes (86.6 ± 2.2% vs 87.6 ± 1.5%, p = 0.48, 2-way ANOVA, Fig. 7H). However, BBG treatment in APP/PS1 neurons co-cultured with APP/PS1 astrocytes did increase ankyrinG intensity (95.2 ± 2.7%, p < 0.0001, 2-way ANOVA) close to the level of WT neurons co-cultured with WT astrocytes. Regarding AIS length, BBG treatment recovered AIS length partially in WT and APP/PS1 neurons in the presence of APP/PS1 astrocytes (p < 0.0001, two-way ANOVA), compared to the absence of effect in WT neurons in the presence of WT astrocytes (p = 0.12), two-way ANOVA) (Fig. 7I). BBG treatment was not able to recover AIS length of APP/PS1 neurons in the presence of WT astrocytes, but instead reduced AIS length (p = 0.0414). These data suggest a role of P2X7 in APP/PS1 astrocytes as a mediator of AIS alterations measured in APP/PS1 mice. Previous studies demonstrated increased P2X7 expression in AD mice model APP/PS1 [51] or Tg2576 [52]. Thus, we checked P2X7 expression in cultured WT and APP/PS1 astrocytes by Western-blot and found around a 20% increased P2X7 expression in APP/PS1 astrocytes (Fig. 7J). Next, we analyzed Adnp expression following astrocytes treatment with BBG (100 nM) for 24 h (Fig. 7K). Adnp expression did not change in WT astrocytes, while BBG treatment in APP/PS1 astrocytes did increase Adnp expression (91.28 ± 10.42% *vs* 58.66 ± 6.07%) compared to WT astrocytes (100 ± 6.32%).

### ADNP-derived NAP peptide recovers ankyrinG decrease induced by APP/PS1 astrocytes

**Fig. 8:**
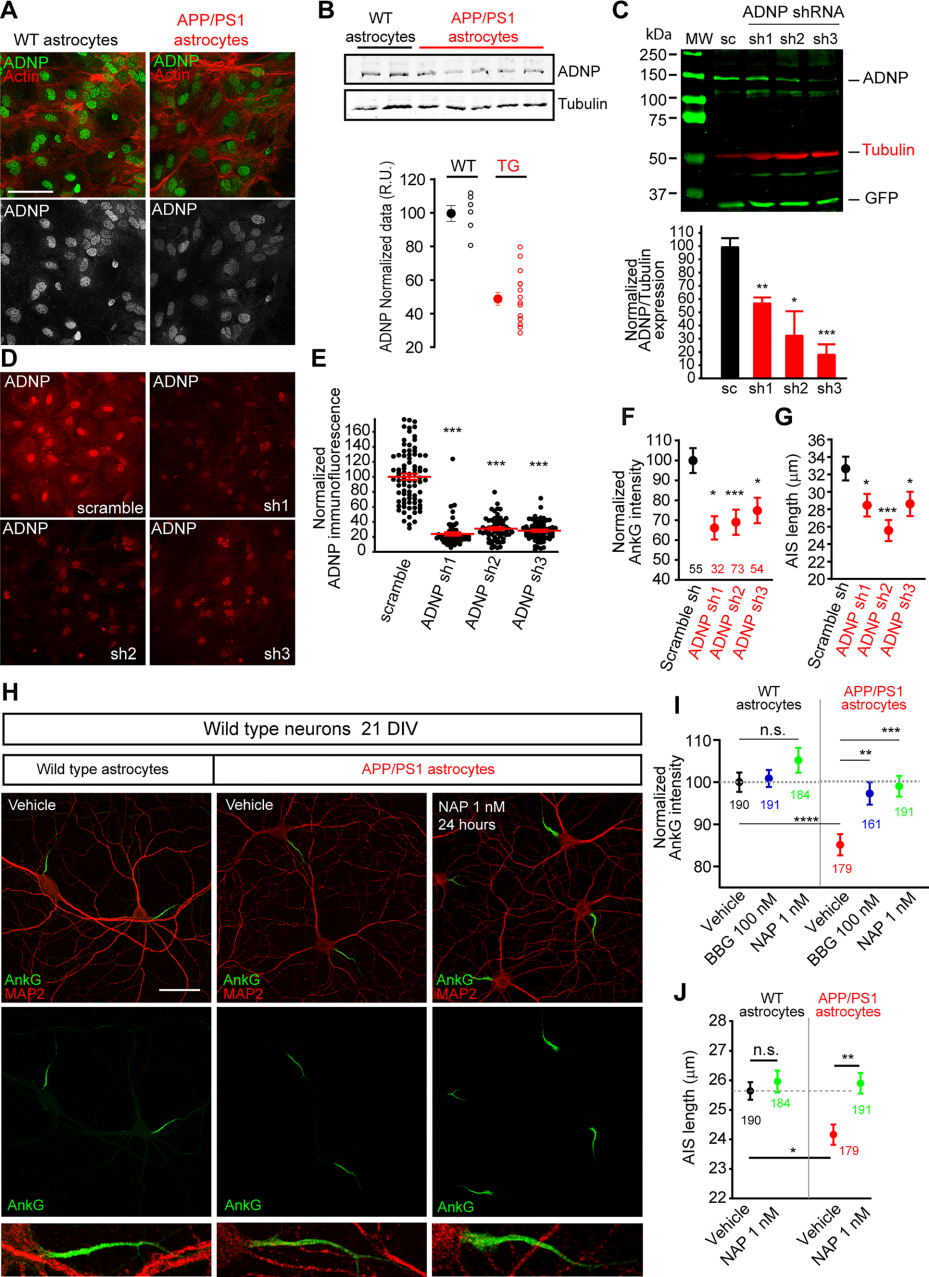
The Adnp-derived peptide, NAP, recovers ankyrinG and AIS length. **A** Representative images of WT and APP/PS1 astrocytes stained with Adnp (green or grey) antibody and phalloidin-Alexa 568 (Actin, red). Scale bar = 50 μm. **B** Normalized Adnp protein expression in WT or APP/PS1 cultured astrocytes and representative Western blot. Adnp was normalized in each sample to their corresponding tubulin intensity. Data were obtained from 6 WT and 13 APP/PS1 independent astrocytes cultures. **C** Western-blot showing Adnp, tubulin and GFP expression in WT astrocytes 7 days after transduction with lentiviral particles expressing scramble shRNA and three different ADNP shRNAs (sh1-3). The graph show the normalized Adnp expression normalized to tubulin expression in 3 independent experiments. *p < 0.05, **p < 0.01, ***p < 0.001, t-test. **D**, **E** Representative images showing ADNP expression in WT astrocytes after 7 days of transduction with a scramble and ADNP shRNAs lentiviral particles. GFP and tubulin stainings of these cells are shown in supplementary Fig. 3. The graph represents the normalized ADNP expression. Data are the mean ± SEM of the indicated number of astrocytes analyzed from 3 independent experiments. ***p < 0.001, one-way ANOVA and Kruskal-Willis post-hoc test with Dunn´s test multiple comparisons. **F**, **G** Normalized ankyrinG intensity and AIS length in WT neurons co-cultured with astrocytes expressing scramble shRNA or ADNP shRNAs 1–3. *p < 0.05, ***p < 0.001, one-way ANOVA and Kruskal-Willis post-hoc test with Dunn´s test multiple comparisons. **H** Representative images of 21 DIV WT neurons cultured in the presence of WT or APP/PS1 astrocytes and treated for 24 h with vehicle or the NAP peptide (10^–9^ M). Neurons were stained with MAP2 (red) and AnkyrinG (green) antibodies. AISs magnifications are shown in the bottom panels. Scale bar = 50 μm. **I**, **J** Normalized ankyrinG (**G**) intensity and AIS length (**H**) in WT cultured neurons in the presence of WT or APP/PS1 astrocytes treated with vehicle or NAP peptide. Data were acquired from 4 independent experiments and around 190 neurons in each condition as shown in the graph. **p < 0.01, ***p < 0.01, ****p < 0.0001. Two-way ANOVA and Bonferroni test for multiple comparisons

Adnp expression is also related to retinoic acid levels [46]. We found a 40% decrease of ADNP mRNA in APP/PS1 astrocytes (Fig. 5) and around a 50% decrease in Adnp protein in APP/PS1 astrocytes (49.2 ± 3.8% *vs* 100 ± 4.7% in WT astrocytes, Fig. 8B). Adnp decrease was also confirmed by immunofluorescence (Fig. 8A). Adnp was mainly expressed in astrocytes nuclei and faintly in the cytoplasm. Adnp cellular distribution corresponds to the one found in previous studies, showing that ADNP is secreted to extracellular medium [53]. However, Adnp levels in astrocytes culture medium were under the detection level of commercial ELISA kits. To confirm the Adnp decrease in astrocytes as a source for AIS alterations, we knockdown Adnp expression using ADNP shRNAs co-expressing GFP, and evaluated Adnp expression by Western-blot (Fig. 8C) and immunofluorescence (Fig. 8D). Western-blot analysis confirmed an Adnp reduction around 39%, 69% and 80% in astrocytes transduced with sh1, sh2 or sh3 ADNP interference RNAs, respectively (Fig. 8C). Immunofluorescence analysis confirmed Adnp reduction, showing a decrease around 70% in all three Adnp shRNAs compared to a control scrambled shRNA (Fig. 8D, E and supplementary Fig. 3). Then, we cultured WT neurons with WT astrocytes previously transduced lentiviral particles expressing scramble shRNA or the three different ADNP shRNAs. A significant AnkyrinG decrease was observed in WT neurons co-cultured with ADNP shRNAs expressing astrocytes (66.2 ± 5.8%, sh1; 69 ± 6.3%, sh2; and 74.8 ± 6.3, sh3, Fig. 8F). AISs were also shorter, showing a reduction of 10%, 20% and 11%, respectively, Fig. 8G). Thus, to decipher the role of secreted Adnp, we took advantage of the neuroprotective effect of an Adnp-derived peptide (NAPVSIPQ). NAP peptide has been shown to provide potent neuroprotection, in vitro and in vivo [54]. To check whether NAP supplementation of extracellular medium might recover ankyrinG density, we cultured WT neurons in the presence of WT or APP/PS1 astrocytes (Fig. 8H). Neurons were treated with vehicle, NAP (1 nM), or BBG (100 nM) at 20 DIV for 24 h. NAP peptide (1 nM) supplementation recovered ankyrinG density (98.9 ± 2.4%) in WT neurons co-cultured with APP/PS1 astrocytes compared to vehicle treatment (85.1 ± 2.5%) or neurons co-culture with WT astrocytes (100 ± 2.3%). Recovery was similar to that observed with BBG treatment (97.2 ± 2.7%). However, NAP treatment did not produce a significant effect in the presence of WT astrocytes (Fig. 8I). Regarding AIS length, NAP treatment recovered AIS length to control levels in the presence of APP/PS1 astrocytes (Fig. 8J). Again, NAP peptide treatment in WT-WT co-cultures did not have any significant effect.

## Discussion

The first initiating cellular mechanisms involved in neuronal function loss leading to AD are still elusive. Here we show that transcriptomic and proteomic alterations of APP/PS1 astrocytes related to retinoic acid and Adnp lead to the loss of ankyrinG and AIS integrity and length in early stages of neuronal development, around P21 or 21 DIV, during the final steps of AIS maturation and the beginning of synaptogenesis. Rdh1 and Aldh1b1 decreased expression may lead to decreased retinoic acid levels which influence the Adnp protein levels expression and increased P2X7 expression. AIS parameters are recovered by impaired retinoic acid degradation, P2X7 inhibition, or Adnp-derived NAP peptide addition.

The AIS is an essential structure that maintains neuronal viability and generates and modulates neuronal excitability [6]. In human post-mortem brain sections, AnkyrinG, the most important protein at the AIS, shows reduced immunofluorescence levels at the AIS [55]. AnkyrinG reduction is also observed by Western-blot analysis in APP/PS1 mice hippocampal tissue at 0, 2, 6, and 8 months [56]. Moreover, AD mouse models show early defects in AIS filtering capacity [17], and there is an absence of AIS plasticity in hiPSCs-derived neurons with a human Tau mutation related to frontotemporal dementia [23].

In our work, AnkyrinG fluorescence quantification at the AIS shows an important AnkyrinG decrease beginning at postnatal day 21 in cortex and hippocampus brain sections. Our analysis using isolated WT or APP/PS1 neurons and astrocytes corroborated this result, confirming the role of APP/PS1 astrocytes in AIS integrity loss and suggesting a role of AIS extrinsic factors derived from astrocytes that contribute to AIS modulation. However, is not possible to exclude lower-intensity additional neuronal intrinsic mechanisms that may influence the AIS indirectly through changes in wild-type astrocytes homeostasis or directly at the AIS. It has been postulated that overexpressed APP can interact with AIS proteins and promote AIS shortening [55]. APP/PS1 hippocampal neurons co-cultured with WT astrocytes showed increased AnkyrinG fluorescence at 6 and 14 DIV that decreased only around 21 DIV. Interestingly, between 15 and 21 DIV in culture or at P21 in the brain cortex, neurons develop dendritic spines [34] and AIS arrives at a mature functional state [57]. We show that AIS in P21 APP/PS1 mice shows an early sharp decline of AnkyrinG fluorescence, suggesting premature aging. Interestingly, a progressive AnkG smooth decay was detected in WT mice from P15 to 16 months in cortical sections, being AnkG levels similar in APP/PS1 and WT mice at 16 months. Molecular changes in the brain cortex are associated with normal aging affecting motor and cognitive capacities maintenance [58]. Moreover, changes in neuronal excitability occur with age in cortical neurons [59]. Actually, neuronal input is closely related to AIS structural plasticity, AIS composition, length or position [8, 60], being P15 to P21 an important stage related to AIS plasticity [61]. Our data show a reduction of AIS length in the hippocampus CA1 region at all ages studied, while in the cortex this reduction is evident at 3 months increasing at 6 months in APP/PS1 mice. This AIS shortening has been described in aged mice of different AD models, including APP_Swe_ mice, R1.40 mice, and APP/TTA mice [15, 55]. Ma et al*.* show AIS shortening in cultured cortical neurons from APP_Swe_ or R1.40 mice [55], similar to our results in 21 DIV WT and APP/PS1 cultured hippocampal neurons in the presence of WT astrocytes. Cultures of cortical neurons shown by Ma et al. contain transgenic non-neuronal cells stained by DAPI. We show that co-culture with APP/PS1 astrocytes reduces AIS length in WT or APP/PS1 hippocampal neurons. As cultured APP/PS1 astrocytes express human Swedish APP mutation (hAPP_Swe_), one possibility is that Aβ oligomers in cultured medium modify AIS length, however, it has been shown that AIS shortening is independent of Aβ oligomers [55]. Moreover, soluble Aβ addition to hippocampal neurons increases AnkG length staining and reduces density in a mechanism associated with microtubules that also affects EB3-GFP mobility and reduces action potential firing [62].

Previous studies have shown that glial cells can contact the AIS [63] or nodes of Ranvier [64], but how these domains are influenced by glial cells contact or secreted factors remains mostly unknown. Different brain cells contain genomic, epigenomic, and proteomic alterations before amyloid plaques can be detected, potentially related to AD onset and development [65]. Thus, which alterations suffer APP/PS1 astrocytes that influence their ability to condition extracellular medium and affect AIS? Analysis by RNAseq of WT and APP/PS1 astrocytes mRNA revealed a significantly reduced mRNA expression in APP/PS1 astrocytes of two enzymes responsible for retinoic acid (RA) synthesis, Rdh1 and Aldh1b1. We confirmed a reduced expression of Aldh1b1 protein and AIS parameters recovery when retinoic acid degradation by CYP26 was inhibited. Even though is not possible to exclude a role of CYP26 inhibition in neurons due to the identification by one study in human adult hippocampal neurons [45], CYP26 expression has not been confirmed in rodent hippocampal neurons, suggesting an effect on astrocytes in our experiments. Indeed, CYP26 knockdown by siRNA in astrocytes increases retinoic acid levels in the culture medium [44]. Astrocytes are the main source of retinoic acid for neurons. Co-cultured experiments and isolated neuronal or astrocytes cultures have shown that retinoic acid levels in isolated neurons are low, but are able to capture astrocytes secreted retinoic acid increasing their levels by ten times [44]. Thus, decreased RA synthesis by APP/PS1 astrocytes may reflect a potential reduction of secreted RA levels affecting neuronal structure and function. Retinoic acid availability is necessary to maintain epithelial cell polarity and tight junctions’ integrity [66], a structure with functional similarities to AIS. Previous studies highlighted the potential role of retinoids in inflammatory diseases and Alzheimer´s disease [43, 67]. Interestingly, RA increases ADNP mRNA expression during P19 cell's neuro/glial differentiation and the absence of Adnp reduces neurite length [46]. ADNP mutations have been detected in AD brains [68], and ADNP is neuroprotective in Alzheimer´s disease models [69, 70], while reduced retinoic acid synthesis has been related to AD and other brain disorders [71]. We found a 50% decrease of Adnp expression in APP/PS1 astrocytes, similar to the 50% reduction of Adnp detected in human serum of early stages AD patients [38]. Moreover, Adnp level reduction in WT astrocytes leads to the same AIS alterations produced by APP/PS1 co-culture. Adnp can shift from the nucleus to cytoplasm, and be secreted to the extracellular medium [53], suggesting that a reduction of secreted Adnp may be a triggering cause of the measured AIS alterations. Adnp-derived peptide, NAP, addition to the medium, recovers AIS parameters. The NAP peptide, derived from Adnp, can recover tau pathology in early stages reducing tau phosphorylation [70, 72]. Adnp expression is significantly reduced in heterozygous MAP6 (Microtubule-associated) mice [73] used as a model of schizophrenia. Interestingly, MAP6 ± mice, treated with NAP, show a substantial amelioration of the cognitive symptoms (differentiation between the novel and familiar objects) and reduced hyperactivity. MAP6 is concentrated at the proximal axon, where stabilizes microtubules and contribute to maintaining voltage-gated sodium channels (Na_v_), which are anchored to the cytoskeleton by AnkG [74] through EB1/EB3 proteins [75], which interacts with Adnp and contributes to microtubules stabilization [37].

Previous studies have shown RA addition contributes to decreasing P2X7 purinergic receptor expression, without affecting P2X7 mRNA expression, and inducing Neuro2a cells differentiation [47]. P2X7 knockout mice crossed with APP/PS1 mice reduce inflammatory response due to glial cells [49]. Our results do not show P2X7 mRNA changes, however, APP/PS1 astrocytes show increased P2X7 expression. P2X7 inhibition recovers AnkG density and partially AIS length in the presence of APP/PS1 astrocytes, both in culture and “in vivo”. Purinergic receptors act as mediators of Ca^2+^-evoked ATP release from astrocytes that regulate axon excitability at AIS and nodes of Ranvier [76]. On the other hand, P2X7 receptor activation is known to impair axonal growth [77] and decreases AIS proteins density compromising AIS integrity and firing frequency [10]. Thus, P2X7-mediated AIS integrity decay may be due to increased P2X7 expression related to reduced RA bioavailability. Moreover, RA addition increases contactin-2 expression [78], an axonal protein expressed at the AIS [79]. Previous results demonstrated the early decline of RA signaling, including synthesis and receptors, in five models of AD and frontotemporal dementia [80].

In conclusion, we have identified transcriptomic and proteomic alterations in APP/PS1 astrocytes related to changes in AIS. Our work points to early alterations of AIS and neuronal function due to altered astrocyte transcriptomic and proteomic expression that may serve as a potential trigger of later Alzheimer´s disease onset. Thus, different genetic or environmental factors early in life that lead to AIS progressive functional deterioration may explain the appearance of Alzheimer´s disease symptoms. WT mice in our study show a progressive soft decay from 1 month of ankyrinG density, leading to levels similar to those observed in APP/PS1 mice at 16 months. Further understanding of the factors that maintain AIS integrity and how integrity is lost during aging will help to generate therapeutic measures to fight brain degeneration with aging. Additional studies are necessary to understand how identified alterations of retinoids, Adnp, and P2X7 are coordinated and lead to early AIS and neuronal function alterations.

## Methods

### Animals

Double-transgenic APP/PS1 mice were purchased from Jackson Laboratories (Bar Harbor; stock no. 005864 (https://www.jax.org/strain/005864)). The strain B6.Cg-Tg (APPSwe, PSEN1dE9) 85Dbo/J overexpresses the human APP gene with the Swedish mutation and exon-9-deleted PSEN1. APP/PS1 mice express a chimeric mouse/human amyloid precursor protein (Mo/HuAPP695swe) and a mutant human presenilin 1 (PS1-dE9).

Mice were housed under constant temperature (22 ± 2 °C) and humidity (50 ± 5%), and a 12:12 h light–dark cycle in a specific-pathogen-free animal facility at Cajal Institute. All animal care and handling strictly followed current Spanish legislation (53/2013, BOE no. 1337) and guidelines of the Council of the European Communities (2010/63/UE). Protocols were previously approved by the CSIC bioethics committee and Community of Madrid (PROEX 083.5–21). Embryos and pups genotypes were confirmed by PCR analysis using three primers: one antisense primer matching sequence within PrP (5′: GTG GAT ACC CCC TCC CCC AGC CTA GAC C), one sense primer specific for the transgene (PS1: 5_: CAG GTG GTG GAG CAA GAT G, APP: 5′: CCG AGA TCT CTG AAG TGA AGA TGG ATG), and a second sense primer specific for the genomic PrP (5′: CCT CTT TGT GAC TAT GTG GAC TGA TGT CGG). Only one band (prion (PrP) gene, used as internal control) was observed in the WT samples, whereas three bands (APP, PS1, and PrP) were observed in the APP/PS1 samples. Treatment of 1-month-old WT and APP/PS1 mice was done by intraperitoneal injection of PBS or BBG (Brilliant Blue G, Sigma-Aldrich, B0770, 50 mg/kg) every 2 days till the age of 3 months.

### Cell cultures

Astrocytes were obtained from individual E17 mice's brain cortex. A piece of brain cortex was kept to genotype each embryo by PCR. Astrocytes genotype was kept blind to the experimenter till data analysis was performed. Each mouse cortex was cut into small pieces, incubated with 0.25% trypsin-1 mg/ml DNAse in Ca^2+^/Mg^2+^ free Hank’s buffered salt solution (HBSS), and dissociated with a 10 ml pipette. The solution was passed through a 70 μm nylon filter, centrifuged at 1200 rpm for 10 min, and resuspended in plating medium (MEM, 10% horse serum, 0.6% glucose, and Glutamax-I). Cells from each embryo were plated at a density of 300.000 cells/ml in several 35 mm cultured plates and maintained for 15–21 DIV till a confluence of around 70%. In some experiments, cells from each embryo were plated at a density of 500.000 cells/ml in 75 mm^2^ culture flasks. Once the genotype was identified, WT or APP/PS1 astrocytes were pulled and plated in 60 mm plates for transcriptomic and proteomic analysis.

For ADNP interference RNA experiments, astrocytes were transduced with scramble or mouse ADNP interference shRNA lentiviral particles (Cat. TL512864V, Origene) for 12 h. Medium was replaced and astrocytes were kept for 4 days before adding neuronal medium for neuronal co-culture or kept for 7 days when used for Western-blotting.

Mouse hippocampal neurons were prepared individually from each embryo as previously described [10, 35]. A piece of brain cortex was kept to genotype each embryo by PCR. Again, the genotype was kept blind to the experimenter till data analysis was performed. Briefly, mice hippocampi were dissected from E17 mice embryos, incubated in a 0.25% trypsin solution in Ca^2+^/Mg^2+^ free Hank’s buffered salt solution (HBSS) and dissociated using fire-polished Pasteur pipettes. Neurons were placed on poly-lysine coated coverslips (1 mg/ml) at a density of 6000 cells/cm^2^ for 2 h in plating medium (MEM, 10% horse serum, 0.6% glucose, and Glutamax-I). Then coverslips containing neurons from one embryo were inverted and transferred to culture dishes containing WT or APP/PS1 astrocytes in neuronal medium (Neurobasal, B27 supplement, and Glutamax-I). 5 μM 1-β-D-arabinofuranosylcytosine (AraC) was added 2 days after to curb glial cells proliferation. Neurons were maintained replacing one-third of the neuronal medium every week.

In some experiments, 21 DIV neurons were treated for the last 24 h with BBG (100 nM, Sigma-Aldrich), the Adnp-derived peptide, NAP (1 nM, TOCRIS 6779), or the last 3 days with the CYP26 inhibitor (50 nM, Sigma-Aldrich SML2092) or their respective vehicle solutions.

### Human-amyloid beta 1–40 and 1–42 measurement

Amyloid levels, secreted by astrocytes, were measured with an Aβ40 or Aβ42 Human ELISA kit (Invitrogen, Paisley, UK) according to the manufacturer's instructions. The absorbance in the plates was read at λ = 450 nm on an Opsys MR Microplate reader (Dynex Technologies, VA, USA).

### Gene expression analysis by RNA-seq and computational data analysis

RNA(n:3) was isolated from cultured astrocytes using TRIzol (Kit RNeasy Mini Kit, Invitrogen), following the manufacturer's instructions. The quality and quantity of each total RNA sample were checked using both a Bioanalyzer and a Nanodrop before proceeding to the RNAseq protocol. 100 ng of total RNA was used to generate barcoded RNA-seq libraries using the NEBNext Ultra II Directional RNA Library preparation kit (New England Biolabs) according to the manufacturer’s instructions. The size of the libraries was checked using the Agilent 2100 Bioanalyzer and the concentration was determined using the Qubit^®^ fluorometer (Life Technologies). Libraries were sequenced on a HiSeq4000 (Illumina) to generate 60 bases of single reads. FastQ files for each sample were obtained using bcl2fastq 2.20 software (Illumina). Sequencing reads were aligned to the human reference transcriptome (GRCh38 v91) and quantified with RSem v1.3.1 (Li and Dewey. 2011). Raw counts were normalized with TPM (Transcripts per Million) and TMM (Trimmed Mean of M-values) methods, transformed into log2 expression (log2(rawCount + 1)), and compared to calculate fold-change and corrected pValue. All data were represented in a Volcano plot. Next, we discarded those genes with log2(FC) values between – 0.3 and + 0.3 and a log10(p-value) < 1.5. The remaining 120 genes were analyzed by ToppGene Suite software to identify clusters of genes associated with biological processes. Most representative genes were represented in a Volcano plot and a heatmap to compare results from each mouse. 

### Immunocytochemistry

Cells were fixed in 4% paraformaldehyde (PFA) / 4% Sucrose for 15 min, washed in phosphate-buffered saline (PBS), treated with 50 mM NH_4_Cl for 10 min, and incubated at RT in blocking buffer for 1 h (0.22% gelatine, 0.1% Triton X-100 in PBS). Primary antibodies were incubated for 1 h diluted in blocking buffer. The primary antibodies used were: mouse monoclonal anti-AnkG (IgG2a, 1:150, clone N106/36, NeuroMab, guinea-pig anti-AnkG, (1:500, SYSY, Cat# 386 004), chicken polyclonal anti-MAP2 (1:5000, Cat# ab5392, Abcam), mouse monoclonal anti β-amyloid (clone 6E10, 1:100, BioLegend), mouse monoclonal anti-acetylated α-tubulin (1:5000, Sigma-Aldrich, T7451), rabbit anti-Adnp (1:500, St John´s STJ22524), rabbit anti-Aldh1b1 (1:100, St John´s STJ22580) and rat anti-GFP (1:500, Nacalai Tesque Inc.). Coverslips were incubated with Alexa-coupled isoform-specific secondary antibodies (1:1000) in blocking buffer for 45 min, and mounted with Fluoromount-G (Southern-Biotech). Images were acquired on a Leica SP5 confocal or a Leica SP8 microscope at a resolution of 1024 × 1024 pixels. Images were analyzed using the ImageJ/Fiji software. Adnp quantification in astrocytes shRNA lentiviral infections was performed designing each cell perimeter based on tubulin staining and quantifying the Adnp channel. GFP was detected in all astrocytes at low levels seven days after infection.

### Western-blot analysis

Protein samples were prepared from WT or APP/PS1 astrocytes cultured to confluence in 75 cm^2^ flasks. The cells were lysed and homogenized in a buffer containing 20 mM HEPES [pH 7.4], 100 mM NaCl, 100 mM NaF, 1% Triton X-100, 1 mM sodium orthovanadate, 10 mM EDTA, and Complete inhibitor protease cocktail (Roche Diagnostics). The proteins were then separated on 8% SDS-PAGE gels and transferred to nitrocellulose membranes. The membranes were incubated overnight at 4 °C with primary antibodies in blocking solution (PBS, 0.2% Tween, 5% non-fat milk). The antibodies used to probe the membranes were: mouse anti-α-tubulin (1∶10.000, Sigma-Aldrich), rabbit anti-Adnp (1:100, St John´s STJ22524), anti-P2X7 (1:250, Abcam AB109054), anti-Aldh1b1 (1:100, St John´s STJ22580) and rat anti-GFP (1:500, Nacalai Tesque, Inc.). Fluorescent secondary antibodies were from LICOR and the signal was analyzed using an Odyssey CLx imaging system (LICOR).

### Immunohistochemistry

Brain hemispheres from P15, P21, P30, and 3, 6- and 16-month-old WT and APP/PS1 mice were quickly removed and immersed in PAF 4% for 1 h, before immersion in 30% sucrose for 24 h at 4 °C. Note that tissues from cardiac perfused animals can be used, but will provide suboptimal staining of AIS markers [81]. Brains were included and frozen in OCT^™^ compound (Tissue-Tek^®^ Sakura, Cat# 4583) and 35 μm brain sections were obtained using a cryostat. Sections from WT and APP/PS1 mice from the same litter and different ages were incubated for 2 h at RT with PBS containing 0.1% TritonX-100 and 10% goat serum, followed by 4 °C overnight incubation with anti-AnkG antibody (IgG2a, 1:150) and anti-amyloid beta antibody (clone 6E10, 1:100) in PBS containing 0.1% TritonX-100 and 1% goat serum. All immunohistochemistry experiments included WT and APP/PS1 1-month-old mice sections. After secondary antibody incubation (1:500) for 2 h at RT, sections were counterstained with bisBenzimide (5 μg/ml, Cat# B2261, Sigma-Aldrich) for nuclei staining and mounted in Fluoromount G. Images were acquired from the CA1 hippocampal region and somatosensory cortex S1 region (Bregma between -1.58 and -1.82, Lateral between 1.04 and 1.92) on a Leica SP5 confocal microscope using Leica DM6000B 40 × 1.25 N.A. oil objective. Sections were imaged in z stacks with a 0.5 μm step size. For quantitative analysis, a Z-projection was obtained in Fiji-ImageJ software.

### AIS parameters quantification

Confocal microscope settings were adjusted to prevent signal saturation and the images were taken in z stacks with a 0.5-μm step size. Cultured neurons images were obtained randomly. All AISs in every brain section image were analyzed. All images from every experiment were obtained using the same settings, standardized in the control samples of each experiment. For quantitative analysis, all coverslips or brain sections in each experiment experienced the same procedures for labeling. AIS was identified by ankyrinG staining. Analysis was performed using Fiji-ImageJ software (NIH) drawing a line along the axon, starting at the soma in cultured neurons and at the beginning of the signal in brain sections. To correct potential background differences between images, mean background was obtained from 6 values surrounding each designed line or region of interest. Correct total fluorescence (CTF) was calculated following the formula: “CTF = Integrated density – (Mean background × Area)” as indicated in ImageJ-Fiji instructions. Data were smoothed every 1 μm using Sigma Plot 14.5 software. AIS start and end positions were determined as described previously [8]. Total fluorescence intensity for each marker was obtained by adding fluorescence intensity values from start to end position after determining AIS length using ankyrinG staining. No significant gap was found between soma and AIS start point in cultured neurons experiments. Data were normalized in each experiment to the value of the mean fluorescence in controls.

### Statistical analysis

All statistical analyses were carried out in GraphPad Prism 8 and Sigmaplot v14.5. The specific test used in each analysis is indicated in the corresponding figure legend. Parametric or non-parametric samples Gaussian distributions were first assessed using the D’Agostino and Pearson omnibus test and Shapiro–Wilk test. Statistical analysis was performed using a two-tailed t-test for two group comparisons: Unpaired t-test for parametric data and a post-hoc Mann–Whitney test for non-parametric data. For multiple group comparisons, a one-way analysis of variance with Tukey post hoc test was used for parametric data, and a post hoc Kruskal–Wallis with a multiple comparison Dunn’s test for non-parametric data. Two-way ANOVA analysis was performed to determine the statistical significance in those experiments including two independent variables, followed by multiple comparisons using Bonferroni test. Data were represented as the mean ± SEM. Differences were considered significant when the P-value was less than 0.05, and represented as: * p < 0.05, ** p < 0.01, *** p < 0.001, or **** p < 0.0001.

## Supplementary Information

Below is the link to the electronic supplementary material.

Supplementary Figure 1. Representative images of somatosensory cortex (upper panels) or hippocampus (bottom panels) brain sections of wild-type or APP/PS1 mice compared in the same litters at 3, 6, and 16 months. Brain sections were stained with antibodies against ankyrinG (AnkG, green) and β-amyloid (6E10, red). Nuclei were stained using bisbenzimide H33342 (blue). Inversed greyscale images show ankyrinG staining at each age. Supplementary Figure 2. (A) Representative images of 21 DIV hippocampal neurons (WT or APP/PS1) cultured in the presence of WT astrocytes. Neurons were stained with antibodies against MAP2 (red), ankyrinG (green), and β-amyloid (6E10, red). (B) Representative images of WT and APP/PS1 cultured astrocytes stained with antibody against β-amyloid (6E10, green). Phalloidin-Alexa 568 (Actin, red) was used to detect actin cytoskeleton and cell morphology, and nuclei were stained with DAPI (blue). (C) WT or APP/PS1 astrocytes stained with anti-β-amyloid antibody (6E10, green) and anti-GM130 antibody (red) to show the expression of β-amyloid in APP/PS1 astrocytes Golgi apparatus. Images show a magnification around the nuclei. Supplementary Figure 3. Representative images of WT astrocytes transduced with lentiviral particles expressing GFP and a scramble shRNA or 3 different ADNP shRNAs (sh1, sh2, and sh3). Astrocytes were fixed 7 days after lentiviral infection and stained with rabbit anti-Adnp, rat anti-GFP, and mouse anti-acetylated tubulin antibodies. Adnp quantification is shown in Figure 8E. Supplementary file1 (PDF 1206 KB)

Supplementary file2 (PDF 270 KB)

## Data Availability

The datasets generated during and/or analysed during the current study are available from the corresponding author on reasonable request. The complete list of genes analyzed and their fold change and significance can be obtained at https://saco.csic.es/index.php/s/EZqD7EEYfPpM3fC

## Material availability

Materials and correspondence to Juan José Garrido (jjgarrido@cajal.csic.es).
