# Supplementary figures and images for: Transcriptomic alterations in APP/PS1 mice astrocytes lead to early postnatal axon initial segment structural changes
Source: Cell Mol Life Sci. 2024 Nov 1;81(1):444. doi: 10.1007/s00018-024-05485-9 (PMC11530419; doi:10.1007/s00018-024-05485-9)

# Supplementary Figure 1

## Cortex

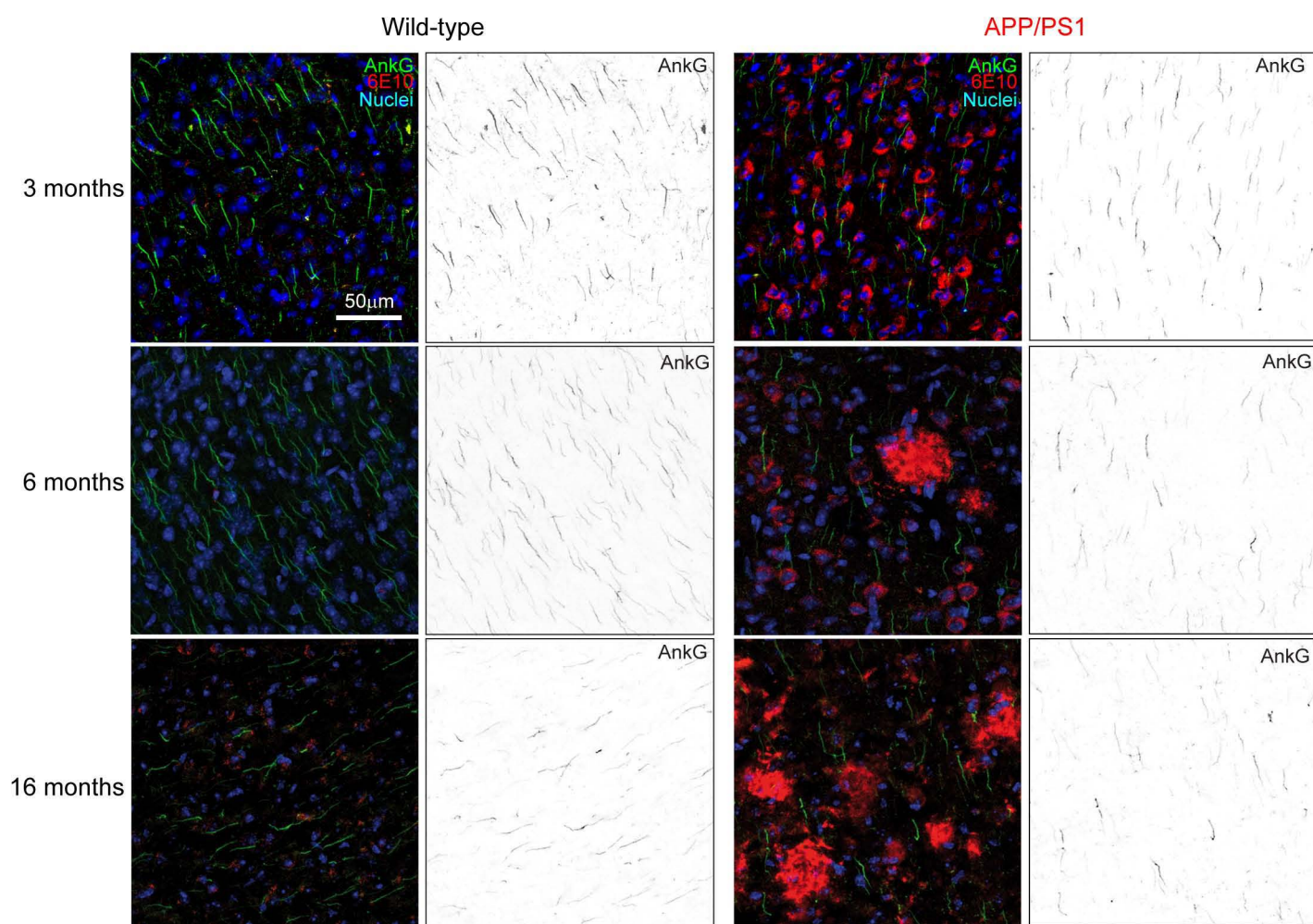

## Hippocampus

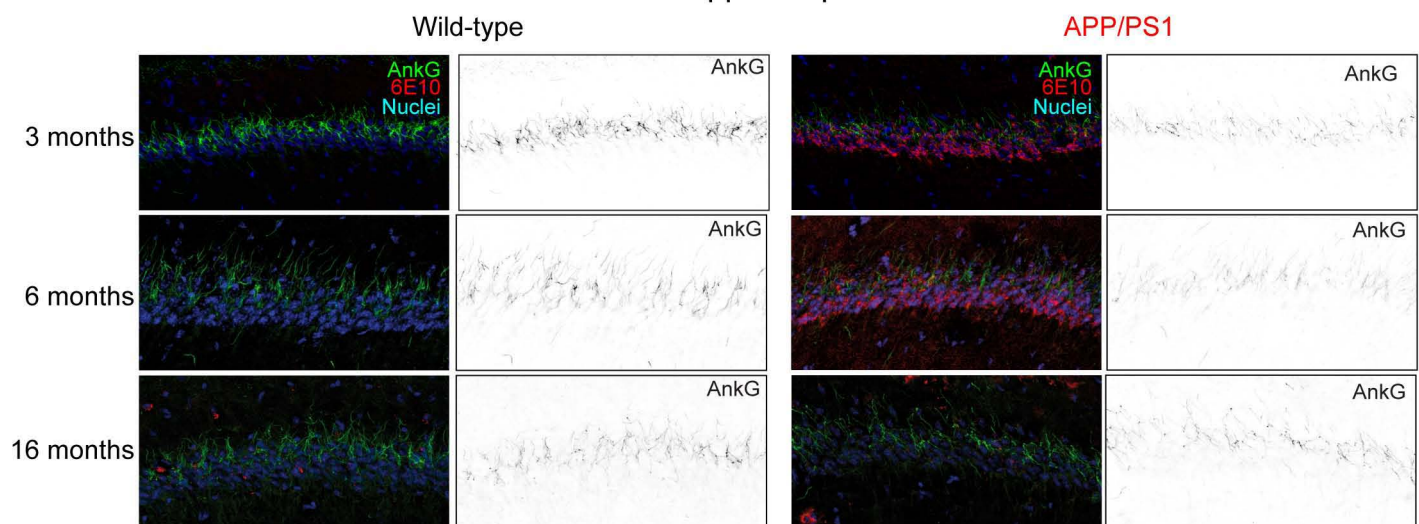

## Supplementary Figure 2

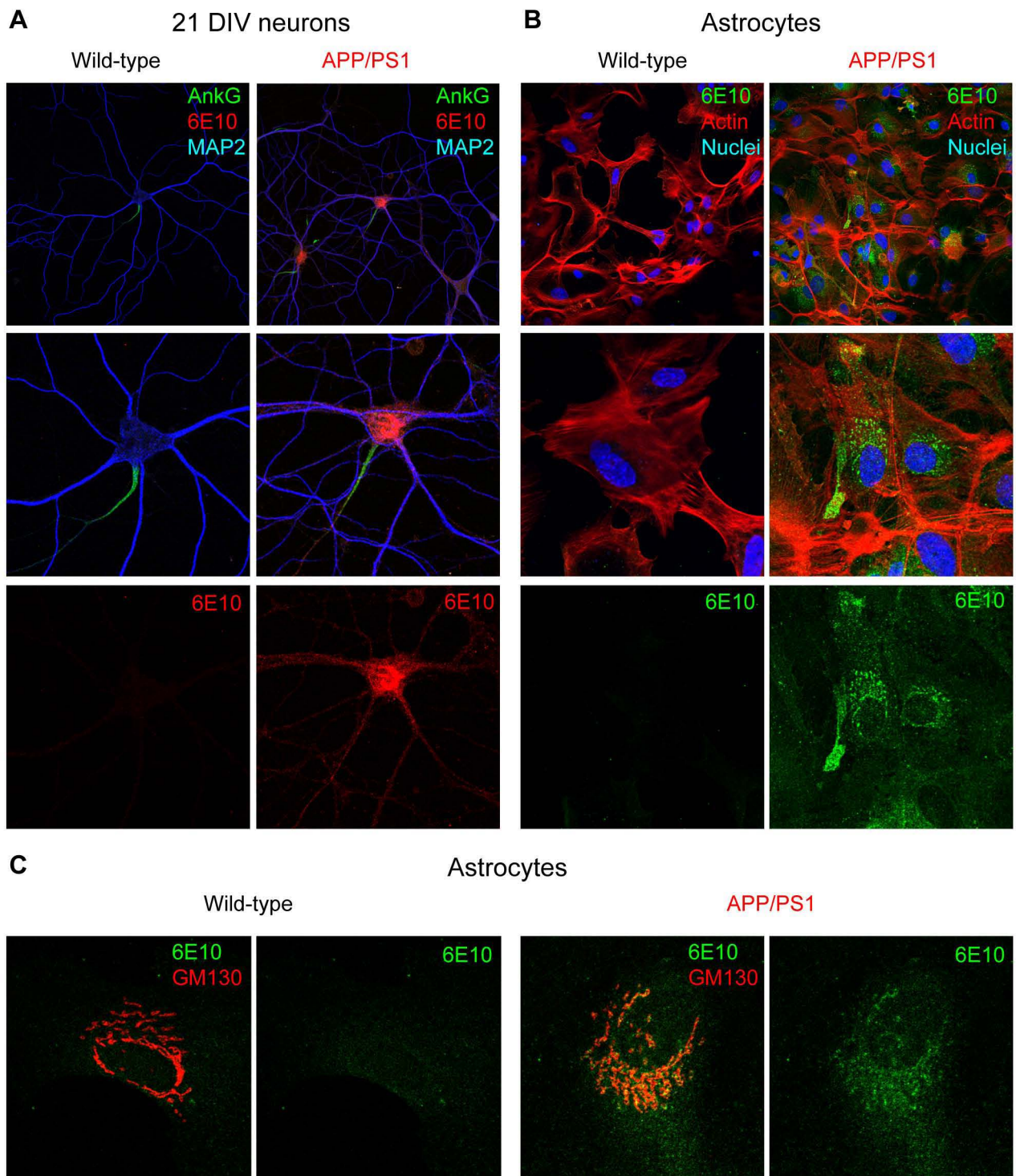

## Supplementary figure 3

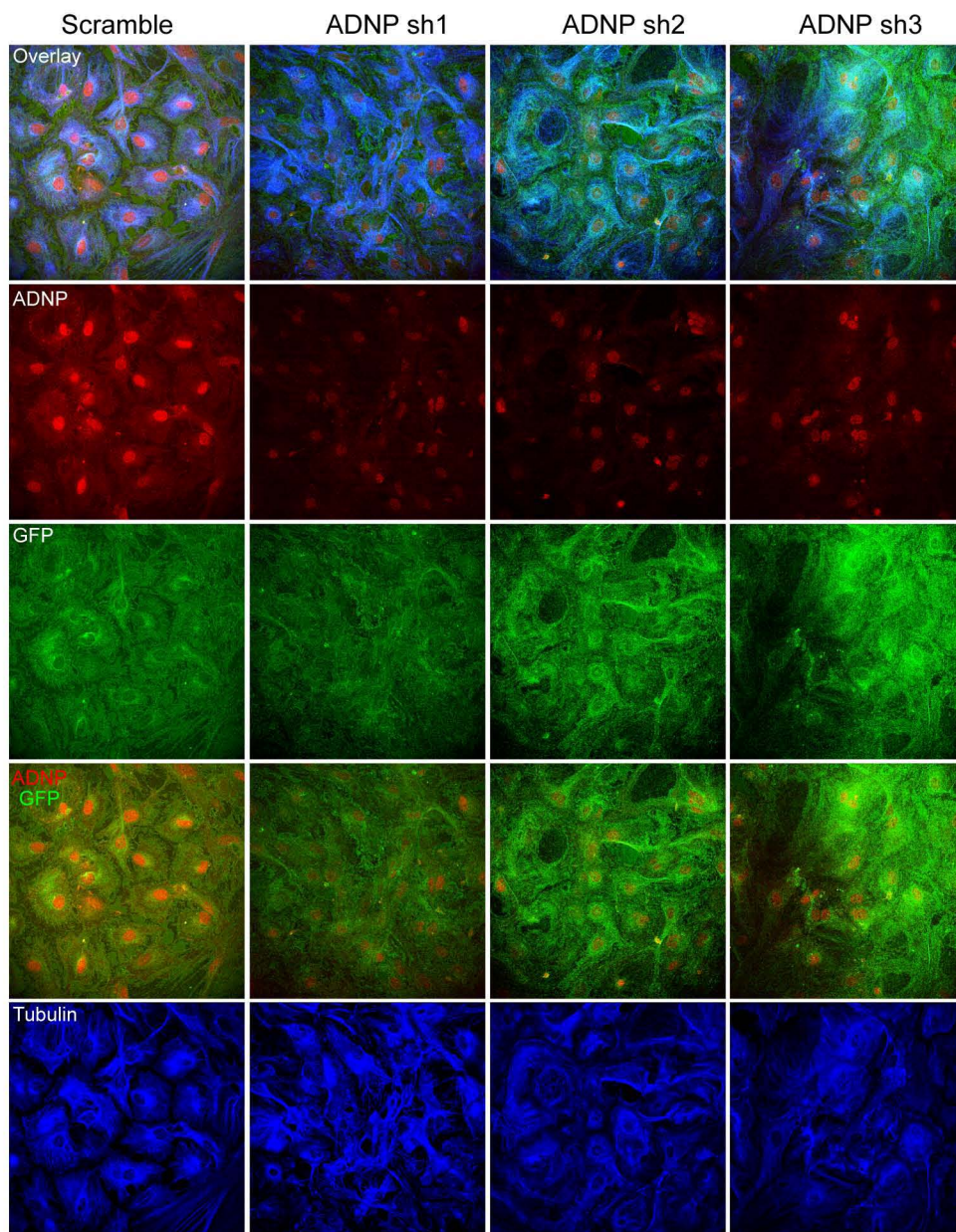

Supplement: Supplementary file 1 — Supplementary Figure 1. Representative images of somatosensory cortex (upper panels) or hippocampus (bottom panels) brain sections of wild-type or APP/PS1 mice compared in the same litters at 3, 6, and 16 months. Brain sections were stained with antibodies against ankyrinG (AnkG, green) and β-amyloid (6E10, red). Nuclei were stained using bisbenzimide H33342 (blue). Inversed greyscale images show ankyrinG staining at each age. Supplementary Figure 2. (A) Representative images of 21 DIV hippocampal neurons (WT or APP/PS1) cultured in the presence of WT astrocytes. Neurons were stained with antibodies against MAP2 (red), ankyrinG (green), and β-amyloid (6E10, red). (B) Representative images of WT and APP/PS1 cultured astrocytes stained with antibody against β-amyloid (6E10, green). Phalloidin-Alexa 568 (Actin, red) was used to detect actin cytoskeleton and cell morphology, and nuclei were stained with DAPI (blue). (C) WT or APP/PS1 astrocytes stained with anti-β-amyloid antibody (6E10, green) and anti-GM130 antibody (red) to show the expression of β-amyloid in APP/PS1 astrocytes Golgi apparatus. Images show a magnification around the nuclei. Supplementary Figure 3. Representative images of WT astrocytes transduced with lentiviral particles expressing GFP and a scramble shRNA or 3 different ADNP shRNAs (sh1, sh2, and sh3). Astrocytes were fixed 7 days after lentiviral infection and stained with rabbit anti-Adnp, rat anti-GFP, and mouse anti-acetylated tubulin antibodies. Adnp quantification is shown in Figure 8E. Supplementary file1 (PDF 1206 KB) [file 18_2024_5485_MOESM1_ESM.pdf]
