# Supplementary material for: Transcriptomic alterations in APP/PS1 mice astrocytes lead to early postnatal axon initial segment structural changes
Source: Cell Mol Life Sci. 2024 Nov 1;81(1):444. doi: 10.1007/s00018-024-05485-9 (PMC11530419; doi:10.1007/s00018-024-05485-9)
Supplement: Supplementary file 2 — Supplementary file2 (PDF 270 KB) [file 18_2024_5485_MOESM2_ESM.pdf]

Supplementary table 1

Transcipt ID	Gene name	Log2(WT)	Log2(TG)	Log2(FC)	pValue
ENSMUSG00000036545	Adamts2	-2,1284	-2,4565	-0,3282	0,0205
ENSMUSG00000044017	Adgrd1	-5,7265	-5,1262	0,6003	0,0301
ENSMUSG00000051149	Adnp	2,4031	2,0038	-0,3993	0,0015
ENSMUSG00000035561	Aldh1b1	-3,0241	-3,3966	-0,3726	0,0095
ENSMUSG00000050022	Amz1	-3,6466	-3,2533	0,3932	0,0316
ENSMUSG00000071847	Apcdd1	-2,3031	-2,6033	-0,3002	0,0081
ENSMUSG00000057346	Apol9a	-2,9358	-2,2685	0,6674	0,0304
ENSMUSG00000068246	Apol9b	-2,5803	-2,9891	-0,4089	0,0001
ENSMUSG00000022892	App	5,9636	6,8445	0,8810	0,0018
ENSMUSG00000028545	Bend5	-5,9641	-6,7341	-0,7700	0,0169
ENSMUSG00000061535	C1qtnf7	-5,1567	-5,8840	-0,7272	0,0208
ENSMUSG00000071037	Camkmt	-2,8291	-2,4551	0,3740	0,0180
ENSMUSG00000034035	Ccdc17	-3,3395	-2,9561	0,3834	0,0240
ENSMUSG00000043953	Ccrl2	-3,6377	-4,3045	-0,6668	0,0293
ENSMUSG00000023274	Cd4	-8,1613	-10,8622	-2,7009	0,0305
ENSMUSG00000061048	Cdh3	-10,4588	-8,1019	2,3569	0,0263
ENSMUSG00000074272	Ceacam1	-4,9261	-4,4698	0,4564	0,0173
ENSMUSG00000047161	Chst9	-9,5481	-7,0415	2,5066	0,0075
ENSMUSG00000058052	Defb35	-7,8942	-8,7395	-0,8453	0,0203
ENSMUSG00000029754	Dlx6	-10,6432	-7,2617	3,3816	0,0008
ENSMUSG00000043753	Dmrta1	-7,7368	-6,2071	1,5297	0,0108
ENSMUSG00000020848	Doc2b	-8,9941	-6,5057	2,4885	0,0118
ENSMUSG00000085576	Dpy19l2	-8,2206	-6,6590	1,5617	0,0201
ENSMUSG00000009145	Dqx1	-4,6186	-6,4764	-1,8578	0,0009
ENSMUSG00000050944	Efcab5	-4,9576	-5,8534	-0,8958	0,0139
ENSMUSG00000018166	Erbb3	-3,8510	-3,4061	0,4449	0,0249
ENSMUSG00000028644	Ermap	-4,7907	-5,5995	-0,8088	0,0005
ENSMUSG00000028773	Fabp3	-5,3173	-6,2712	-0,9539	0,0154
ENSMUSG00000043068	Fam89a	-8,4741	-7,3858	1,0883	0,0290
ENSMUSG00000000392	Fap	-7,3289	-9,5871	-2,2582	0,0150
ENSMUSG00000027344	Fsip1	-4,5662	-5,4754	-0,9092	0,0139
ENSMUSG00000009633	G0s2	-4,7549	-3,9941	0,7609	0,0027
ENSMUSG00000093805	Gal3st2b	-6,5723	-7,3125	-0,7402	0,0231
ENSMUSG00000030498	Gas2	-2,3670	-2,0115	0,3554	0,0061
ENSMUSG00000009108	Gnat2	-6,0923	-5,2762	0,8161	0,0027
ENSMUSG00000091931	Gon7	-2,4393	-2,0424	0,3969	0,0037
ENSMUSG00000046856	Gpr1	-7,8652	-5,9567	1,9085	0,0126
ENSMUSG00000040125	Gpr26	-5,3483	-7,7395	-2,3912	0,0219
ENSMUSG00000049608	Gpr55	-8,8942	-7,9033	0,9909	0,0194
ENSMUSG00000030209	Grin2b	-1,9429	-2,2466	-0,3038	0,0122
ENSMUSG00000046182	Gsg1l	-5,7130	-7,6613	-1,9482	0,0249
ENSMUSG00000041624	Gucy1a2	-0,6918	-1,0458	-0,3539	0,0031
ENSMUSG00000073405	H2-T-ps	-3,9628	-3,5297	0,4331	0,0070
ENSMUSG00000032338	Hcn4	-6,3190	-5,3065	1,0125	0,0023
ENSMUSG00000045662	Henmt1	-7,8942	-10,8622	-2,9680	0,0034
ENSMUSG00000069267	Hist1h3b	-3,7626	-4,0685	-0,3059	0,0223
ENSMUSG00000085347	Hk1os	-4,6701	-5,6934	-1,0233	0,0044
ENSMUSG00000001014	Icam4	-5,9111	-6,9094	-0,9982	0,0104

ENSMUSG00000028037	Ifi44	-1,7368	-1,3744	0,3624	0,0081
ENSMUSG00000005672	Kit	-1,6572	-2,0724	-0,4151	0,0200
ENSMUSG00000058488	Kl	-8,9328	-7,1019	1,8308	0,0279
ENSMUSG00000029195	Klb	-6,1495	-7,6613	-1,5118	0,0073
ENSMUSG00000109764	Klkb1	-4,6309	-5,2154	-0,5846	0,0035
ENSMUSG00000030124	Lag3	-5,7482	-6,6999	-0,9517	0,0202
ENSMUSG00000039706	Ldb2	-5,0483	-4,3154	0,7329	0,0185
ENSMUSG00000031637	Lrp2bp	-3,6061	-4,1942	-0,5881	0,0281
ENSMUSG00000086450	Macrod2os1	-5,4524	-4,7130	0,7394	0,0125
ENSMUSG00000028979	Masp2	-3,9149	-4,6722	-0,7573	0,0095
ENSMUSG00000038528	Mfsd4b5	-8,4459	-6,8648	1,5810	0,0312
ENSMUSG00000025389	Mip	-7,2774	-10,3561	-3,0787	0,0312
ENSMUSG00000004996	Mri1	-2,4913	-2,0854	0,4059	0,0114
ENSMUSG00000025930	Msc	-10,6432	-7,7395	2,9037	0,0194
ENSMUSG00000005493	Msh4	-6,4274	-7,7288	-1,3013	0,0258
ENSMUSG00000020181	Nav3	-2,1941	-2,6054	-0,4113	0,0088
ENSMUSG00000025723	Nmb	-4,4232	-4,9746	-0,5515	0,0245
ENSMUSG00000019865	Nmbr	-6,2487	-5,6767	0,5720	0,0105
ENSMUSG00000037349	Nudt22	-2,1740	-1,7801	0,3940	0,0263
ENSMUSG00000091531	Olfr102	-5,9309	-4,9771	0,9538	0,0231
ENSMUSG00000075102	Olfr1221	-8,8942	-7,3858	1,5084	0,0032
ENSMUSG00000095809	Olfr1290	-6,6815	-9,5871	-2,9056	0,0005
ENSMUSG00000095917	Olfr740	-8,8368	-7,0481	1,7887	0,0247
ENSMUSG00000043331	Olfr975	-6,9185	-5,6190	1,2996	0,0083
ENSMUSG00000027584	Oprl1	-4,3910	-5,4207	-1,0297	0,0198
ENSMUSG00000049112	Oxtr	-5,2057	-5,6401	-0,4344	0,0081
ENSMUSG00000092534	Pagr1b	-3,6528	-3,2171	0,4357	0,0076
ENSMUSG00000037034	Pax1	-3,6528	-3,2171	0,4357	0,0303
ENSMUSG00000020098	Pcbd1	-2,8850	-3,2008	-0,3159	0,0026
ENSMUSG00000045657	Pcdhb10	-4,9412	-5,6793	-0,7381	0,0057
ENSMUSG00000063687	Pcdhb5	-3,5510	-4,0832	-0,5323	0,0064
ENSMUSG00000051242	Pcdhb9	-3,4167	-3,8785	-0,4618	0,0096
ENSMUSG00000103081	Pcdhgb8	-5,1952	-4,0506	1,1446	0,0242
ENSMUSG00000097111	Peak1os	-10,4588	-7,7288	2,7301	0,0287
ENSMUSG00000070366	Plpp4	-6,5840	-9,8622	-3,2782	0,0029
ENSMUSG00000017417	Plxdc1	-5,2394	-4,6135	0,6260	0,0171
ENSMUSG00000029759	Pon3	-2,6610	-2,2376	0,4233	0,0066
ENSMUSG00000029410	Ppef2	-6,2799	-5,1385	1,1415	0,0296
ENSMUSG00000079037	Prnp	5,5205	6,6149	1,0945	0,0016
ENSMUSG00000093629	Prox2os	-5,9234	-5,2322	0,6912	0,0183
ENSMUSG00000090273	Prr22	-8,5481	-6,6539	1,8942	0,0085
ENSMUSG00000074359	Psg23	-9,5481	-6,5356	3,0125	0,0294
ENSMUSG00000072423	Psmb11	-6,4306	-4,9794	1,4512	0,0192
ENSMUSG00000031504	Rab20	-5,5799	-5,0129	0,5670	0,0309
ENSMUSG00000079657	Rab26	-3,4971	-3,1749	0,3222	0,0298
ENSMUSG00000025170	Rab40b	-3,6313	-3,1508	0,4805	0,0279
ENSMUSG00000087397	Rapgef3os2	-5,2224	-4,4445	0,7779	0,0081
ENSMUSG00000089789	Rdh1	-5,9015	-6,8191	-0,9176	0,0019
ENSMUSG00000023140	Reg2	-4,6848	-5,4111	-0,7264	0,0302
ENSMUSG00000031438	Rnf128	-4,1648	-3,3281	0,8366	0,0119

ENSMUSG00000094772	Rpl29-ps2	-5,2162	-6,7772	-1,5610	0,0023
ENSMUSG00000026121	Sema4c	-1,3047	-1,6225	-0,3178	0,0087
ENSMUSG00000001508	Sgca	-3,6013	-4,0497	-0,4485	0,0221
ENSMUSG00000073758	Sh3d21	-4,5947	-4,2257	0,3690	0,0209
ENSMUSG00000038738	Shank1	-3,5647	-4,3199	-0,7552	0,0281
ENSMUSG00000033114	Slc35d2	-2,7710	-2,3736	0,3974	0,0002
ENSMUSG00000068323	Slc4a5	-9,5481	-7,5827	1,9654	0,0102
ENSMUSG00000020838	Slc6a4	-6,5000	-8,1019	-1,6020	0,0066
ENSMUSG00000017002	Slpi	-10,4588	-7,3858	3,0731	0,0048
ENSMUSG00000020027	Socs2	-4,5888	-5,1634	-0,5746	0,0088
ENSMUSG00000011751	Sptbn4	-3,5834	-4,0485	-0,4651	0,0070
ENSMUSG00000024172	St6gal2	-4,5928	-5,5659	-0,9731	0,0068
ENSMUSG00000021071	Trim9	-0,9464	-1,3343	-0,3879	0,0247
ENSMUSG00000021569	Trip13	-3,3054	-3,7215	-0,4160	0,0135
ENSMUSG00000035186	Ubd	-10,4588	-8,1019	2,3569	0,0263
ENSMUSG00000055891	Ubl4b	-10,6432	-8,1019	2,5413	0,0182
ENSMUSG00000063626	Unc5d	-2,8360	-2,2867	0,5493	0,0048
ENSMUSG00000086040	Wipf3	-5,8625	-7,9033	-2,0408	0,0122
ENSMUSG00000093452	Zfhx2os	-8,1844	-6,4176	1,7668	0,0216
ENSMUSG00000044519	Zfp488	-2,8008	-3,2986	-0,4978	0,0294
ENSMUSG00000034429	Zfp707	-2,0356	-2,3405	-0,3049	0,0103
